# Crosstalk and Signaling Switches in Mitogen-Activated Protein Kinase Cascades

**DOI:** 10.3389/fphys.2012.00355

**Published:** 2012-09-18

**Authors:** Dirk Fey, David R. Croucher, Walter Kolch, Boris N. Kholodenko

**Affiliations:** ^1^Systems Biology Ireland, University College DublinDublin, Ireland

**Keywords:** dynamic model, bistability, JNK mitogen-activated protein kinases, Akt (PKB), dual specificity phosphatase

## Abstract

Mitogen-activated protein kinase (MAPK) cascades control cell fate decisions, such as proliferation, differentiation, and apoptosis by integrating and processing intra- and extracellular cues. However, similar MAPK kinetic profiles can be associated with opposing cellular decisions depending on cell type, signal strength, and dynamics. This implies that signaling by each individual MAPK cascade has to be considered in the context of the entire MAPK network. Here, we develop a dynamic model of feedback and crosstalk for the three major MAPK cascades; extracellular signal-regulated kinase (ERK), p38 mitogen-activated protein kinase (p38), c-Jun N-terminal kinase (JNK), and also include input from protein kinase B (AKT) signaling. Focusing on the bistable activation characteristics of the JNK pathway, this model explains how pathway crosstalk harmonizes different MAPK responses resulting in pivotal cell fate decisions. We show that JNK can switch from a transient to sustained activity due to multiple positive feedback loops. Once activated, positive feedback locks JNK in a highly active state and promotes cell death. The switch is modulated by the ERK, p38, and AKT pathways. ERK activation enhances the dual specificity phosphatase (DUSP) mediated dephosphorylation of JNK and shifts the threshold of the apoptotic switch to higher inputs. Activation of p38 restores the threshold by inhibiting ERK activity via the PP1 or PP2A phosphatases. Finally, AKT activation inhibits the JNK positive feedback, thus abrogating the apoptotic switch and allowing only proliferative signaling. Our model facilitates understanding of how cancerous deregulations disturb MAPK signal processing and provides explanations for certain drug resistances. We highlight a critical role of DUSP1 and DUSP2 expression patterns in facilitating the switching of JNK activity and show how oncogene induced ERK hyperactivity prevents the normal apoptotic switch explaining the failure of certain drugs to induce apoptosis.

## 1 Introduction

A hallmark of cancer is dysregulation of pivotal cell fate decisions leading to aberrant proliferation and reduced apoptosis (Hanahan and Weinberg, 2011). Healthy cell fate decisions depend on a proper sensing of the cell’s intra- and extracellular environment in a process called signal transduction (Kholodenko et al., 2010). The signals are sensed by receptors that bind their cognate extracellular ligands, resulting in conformational changes that trigger the formation of multi-protein complexes and subsequent activation of GTPases and kinases (Lemmon and Schlessinger, 2010). Hereby, one receptor usually activates several downstream pathways. Main transducers are MAPK cascades, which consist of a linear array of three kinases where a GTPase activates a MAPK kinase kinase (MAPKKK; MAP3K), which phosphorylates and activates a MAPK kinase (MAPKK; MAP2K), which in turn activates a MAPK that delivers the main pathway output by phosphorylation of multiple substrates (Kolch, 2005; Dhillon et al., 2007). MAPKs and MAP2Ks are activated by dual phosphorylation, which can confer switch like properties to the activation kinetics (Kholodenko, 2000). Sometimes a MAP4K is intercalated between the GTPase and the MAP3K. A particular cell fate cannot be attributed to the activity of a single protein in isolation, but rather depends on the context, including the temporal patterns of activation and the regulatory feedback structures within the signaling network (Kholodenko, 2006; Kholodenko et al., 2010; Nakakuki et al., 2010). Because of this complexity, the function of cellular signaling often eludes a naive intuitive understanding, thus calling for the use of mathematical modeling and analysis (Kitano, 2002, 2010; Ireton et al., 2009). Whereas others approach the problem from a less mechanistic viewpoint using regression (Miller-Jensen et al., 2007) or Boolean and semi-logic models (Saez-Rodriguez et al., 2009, 2011), we focus on dynamic models using ordinary differential equations.

Dynamic modeling has played a key role in understanding how signaling via the ERK cascade regulates cell fate (Kholodenko et al., 2010; Sturm et al., 2010). A classic example is growth factor signaling in Rat Pheochromocytoma (PC12) cells, where treatment with epidermal growth factor (EGF) or nerve growth factor (NGF) activates the same signaling cascade (the RAF/MEK/ERK cascade) but has different effects on cell fate. EGF causes transient activation of ERK and proliferation due to negative feedback, whereas NGF causes sustained ERK activation and differentiation due to positive feedback (Santos et al., 2007; von Kriegsheim et al., 2009). Similarly, the stress activated MAPKs JNK and p38 mediate diverse cellular responses. For example, growth factor induced, transient activation of JNK promotes cell survival and proliferation, whereas stress induced, prolonged JNK activity promotes growth arrest and cell death (Ventura et al., 2006). However, the mechanistic details of how this switch is generated and the factors determining the shift from proliferative to apoptotic JNK signaling are poorly understood, and mathematical modeling and analysis is largely lacking for stress activated kinases (Bagowski and Ferrell, 2001; Wagner and Nebreda, 2009).

Here, we provide a dynamic model of feedback and crosstalk for the three major MAPKs (ERK, p38, JNK) and protein kinase B (AKT) signaling. The model incorporates mechanistic details of positive feedback from JNK to its own MAP3Ks and negative crosstalk from and to other pathways. Using mathematical analysis, the model is used to decipher how JNK switches from proliferative to apoptotic signaling and how that switch is regulated by pathway crosstalk.

## 2 Results

We present a dynamic model of multiple MAPK cascade interactions featuring a JNK positive feedback loop that generates a proliferative-apoptotic switch. Further, we present a detailed analysis of factors controlling the dynamic properties of the JNK switch, with a particular focus on feedback loops and crosstalk.

### 2.1 Nominal model of MAPK interactions

**Figure 1:**
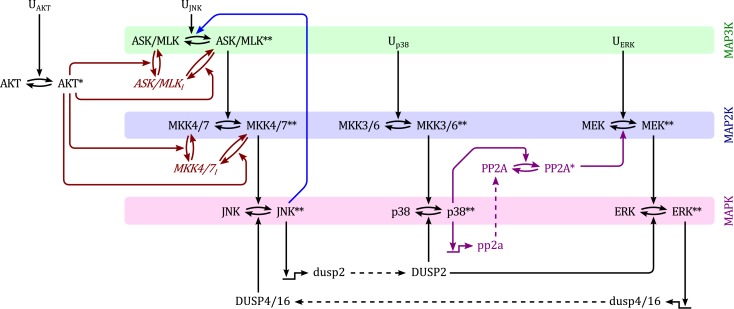
**Scheme of the nominal MAPK interaction model**. For simplicity of illustration, the double phosphorylation of MAP(K) kinases are depicted in single steps and the three inactive forms of ASK/MLK and MKK4/7 are lumped into one component. *U_i_* denote inputs that are modeled as time-dependent functions (not modeled with differential equations). Black: nominal cascades; Blue: positive feedback from JNK to its own MAP3Ks; Red: negative crosstalk from AKT to JNK signaling; Purple: negative crosstalk from p38 to ERK signaling only occurring in non-transformed cells. Lower- and upper-case letter indicate mRNAs and proteins, respectively. Single and double asterisk indicate single- and double-phosphorylated active forms, respectively.

Although MAPK signaling cascades have been studied extensively, the connectivity of MAPK systems is not completely understood. MAPKs feature several isoforms, a high number of inputs in the form of different GTPases and protein kinases, several scaffolding proteins that channel incoming signals into different pathways and a variety of phosphatases that modulate MAPK activation dynamics. Thus, depending on the expression and activity states of these proteins, MAPK connections change between cell types and in response to pathological aberrations. In order to analyze the kinetic behavior and regulation of MAPK cascades we constructed a model which represents a core network of MAPK interactions based on the available literature. The topology of this model is depicted in Figure 1.

Generally, MAPK systems are arranged in three tiered cascades consisting of MAPKs (lowest tier), MAPK kinases (MAP2Ks, second tier), and MAPK kinases kinases (MAP3Ks, top tier). Activation of the kinases in each tier is modeled with double phosphorylation cycles as described in *Material and Methods* (Sec. 5), in which the upstream kinase acts as the enzyme catalyzing the phosphorylation and therefore activation of the downstream kinase. Complementing the classical cascades, the model features several crosstalks and feedbacks (Table 1). First, JNK phosphorylates and activates its own MAP3K (Schachter et al., 2006; Furuhata et al., 2009), generating a positive feedback loop. Second, p38 inhibits ERK activity by enhancing MEK dephosphorylation either via transcriptional upregulation or phosphorylation of protein phosphatase 2 (PP2A; Westermarck et al., 2001; Li et al., 2003; Liu and Hofmann, 2004; Grethe and Pörn-Ares, 2006; Junttila et al., 2008). Third, ERK inhibits JNK via induction of dual specificity phosphatases (DUSPs) catalyzing the dephosphorylation of JNK (Paumelle et al., 2000; Monick et al., 2006). Finally, AKT inhibits JNK activity by phosphorylating inhibitory sites in the JNK-MAP3Ks and -MAP2Ks (Kim et al., 2001; Park et al., 2002; Barthwal et al., 2003).

**Table 1:** **Crosstalks and feedbacks in the nominal MAPK interaction model**.

Interaction			Mechanism	Comments	Reference
JNK	→	ASK1	Oligomerization and auto-phosphorylation	Via JNK induced ROS production (WEHI-231)	Furuhata et al. (2009)
	→	MLK3	Phosphorylation	Direct JNK mediated phosphorylation (HEK 293, Hela, MCF-7)	Schachter et al. (2006)
p38	⊣	ERK	Upregulation of PP2A	Only in non-immortalized, non-transformed cells	Westermarck et al. (2001), Li et al. (2003), Liu and Hofmann (2004), Grethe and Pörn-Ares (2006), Junttila et al. (2008)
ERK	⊣	JNK	Induction of DUSP4 and DUSP16	MDCK epithelial cells, human alveolar macrophages	Paumelle et al. (2000), Monick et al. (2006)
AKT	⊣	ASK1	Phosphorylation at S83	HEK293, L929	Kim et al. (2001)
	⊣	MLK3	Phosphorylation at S674	HepG2	Barthwal et al. (2003)
	⊣	MKK4	Phosphorylation at S78	HEK293T	Park et al. (2002)
JNK	⊣	ERK, p38	Induction of DUSPs via Jun gene transcription, DUSP2 is the speculative isoform assumed in the model	What DUSP isoforms are involved is unclear; DUSP1, 4, 6 were not detected in COS-7	Chu et al. (1996), Black et al. (2002), Shen et al. (2003), Stepniak et al. (2006), Junttila et al. (2008), Peng et al. (2009)

*The list comprises core interactions which are supposedly conserved between cell lines, but with the p38-ERK crosstalk being restricted to non-transformed cells. The comments column indicates the experimental system (cell lines) used to identify the links*.

In the following section we review experimental evidence for each crosstalk mechanism and show how they are implemented in the dynamic model. Finally, we explore the intricate kinetic behavior and dynamics of three MAPK cascades.

#### 2.1.1 JNK positive feedback

Several studies support the idea of a JNK positive feedback loop on the systems level. For example, JNK positive feedback was critical for a proper stress response of Xenopus oocytes (Bagowski and Ferrell, 2001). In mammalian cells, JNK exhibited all-or-none responses on the single cell level after treatment with anisomycin or sorbitol (Bagowski et al., 2003), and a positive feedback loop was suggested (Bagowski et al., 2003; Xiong and Ferrell, 2003). On the population level, these all-or-none responses manifest highly ultrasensitive behavior with apparent Hill coefficients as high as 9 or 10 (Table 2), which is consistent with the presence of a positive feedback loop, which increases the degree of ultrasensitivity (Bagowski et al., 2003).

**Table 2:** **Ultrasensitivity of the JNK response to stress in mammalian cell populations (Bagowski et al., 2003)**.

Stimulus	Apparent Hill Coefficient		
	HeLa	HEK293	Jurkat
Sorbitol	9	8	4
Anisomycin	10	4	3

The literature contains considerable evidence supporting the existence of positive feedback from JNK to its own MAP3Ks, in particular to mixed linage kinases (MLK) and apoptosis regulated kinases (ASK; Xu and Cobb, 1997; Phelan et al., 2001; Ventura et al., 2004; Schachter et al., 2006; Furuhata et al., 2009). For example, in HEK 293, Hela and MCF-7 cells, JNK phosphorylated MLK3 directly at sites in the COOH-terminal region, which resulted in the redistribution of MLK3 to triton-insoluble membrane microdomains, increased phosphorylation of the activation loop and increased MLK3 activity (Schachter et al., 2006). Similarly in COS-7 cells, JNK phosphorylated the C-terminal domain of MLK2, which was required for MLK2-induced apoptosis (Phelan et al., 2001). Further, JNK phosphorylated a MEKK1 fragment *in vitro* and coimmunoprecipitated with MEKK1 in HEK 293 cells (Xu and Cobb, 1997). MEKK1 is a MAP3K for the JNK pathway, which depending on its phosphorylation status, also can act as a scaffold for the MEKK1-MKK4-JNK pathway (Gallagher et al., 2002).

Another, more indirect route of JNK feeding back to its own MAP3Ks involves the production of reactive oxygen species (ROS). In fibroblasts, JNK produced ROS after TNF treatment, in a process that did not involve gene transcription and was inhibited by NF-κB (Ventura et al., 2004). Interestingly, several signaling pathways connect ROS to JNK activation, suggesting a JNK-ROS positive feedback loop (Shen and Liu, 2006). ASK1 in particular is readily activated by ROS, whereby ROS induces the dissociation of ASK from internal inhibitors such as thioredoxin or 14-3-3 proteins, finally resulting in ASK1 oligomerization and phosphorylation of its activation loop (Saitoh et al., 1998; Goldman et al., 2004; Shen and Liu, 2006). In fact, such a ROS dependent positive feedback loop has been reported in WEHI-231 mouse B lymphoma cells, where JNK activity produced hydrogen peroxide (H_2_O_2_) which, in turn, activated ASK1 (Furuhata et al., 2009).

The molecular mechanisms of MAP3K activation are quite complex. For example, MLK3 activation involves GTPases binding, translocation to the membrane, dimer- or oligomerization, and activation loop phosphorylation of MLK3 at Thr2277 and Ser281 (Schachter et al., 2006). Neglecting this complexity, and in concordance with earlier models in the literature, we model the activation of MAP3Ks as a phosphorylation process catalyzed by its inputs (Kholodenko, 2000; Kholodenko et al., 2010). In the model, different JNK-MAP3Ks are lumped into one ASK/MLK component. The activation of this component is modeled as a double phosphorylation cycle with two inputs, representing the activity of upstream GTPases *u*_3_ and active JNK (see Figure 1). Although our model simplifies the involved molecular events, it captures the main feature of MAP3K activation, namely the phosphorylation of two conserved residues in the activation loop.

#### 2.1.2 p38 inhibits ERK signaling in non-transformed cells

Generally, ERK activity promotes survival. The suppression of this activity by p38 is critical for induction of apoptosis in non-transformed cells and PP2A mediates this effect (Junttila et al., 2008). In particular, p38 mediated dephosphorylation of MEK was necessary for arsenite induced apoptosis in human skin fibroblasts (HSF) and rat primary neurons (CGN), but not in transformed and tumorigenic cell lines (HeLa, Jurkat, K562, HT-1080, WM266-4, A2058; Li et al., 2003). Further, PP2A mediated this p38-MEK negative crosstalk and was required for both cytokine and stress induced apoptosis in human endothelium cells and rat cardiac ventricular myocytes, respectively (Liu and Hofmann, 2004; Grethe and Pörn-Ares, 2006).

How p38 regulates PP2A is uncertain. PP2A is a heterotrimer composed of a scaffold, a catalytic subunit and different regulatory subunits. Its catalytic activity can be regulated on several levels, including assembly of the heterotrimer with different regulatory subunits, and both phosphorylation or methylation of the catalytic subunit (Janssens and Goris, 2001; Nguyen et al., 2012). Because the mechanism by which p38 upregulates PP2A activity is uncertain, and because the system dynamics depend on this mechanism, our model implements two possibilities, each on opposite ends of the dynamic spectrum: slow upregulation via gene transcription of the regulatory subunit, and fast activation via phosphorylation of the catalytic subunit.

#### 2.1.3 ERK inhibits JNK

ERK signaling strongly induces several DUSPs, some of which negatively regulate JNK activity. For example, DUSP4 is readily induced in response to several growth factors (Legewie et al., 2008; Cagnol and Rivard, 2012) and stabilization of DUSP16 by ERK mediated phosphorylation at Ser-446 was observed in both COS-7 (fibroblastic) and Hela cells (Katagiri et al., 2005). Further, ERK enhanced JNK dephosphorylation by induction of DUSP4 in Madin-Darby canine kidney (MDCK) epithelial cells (Paumelle et al., 2000) and ERK inhibition in human alveolar macrophages (which are part of the immune system in lung) reduced DUSP16 levels, resulting in increased JNK phosphorylation (Monick et al., 2006). Together, these data indicate that the DUSP4/16 mediated ERK-JNK crosstalk is conserved between cell lines (epithelial, fibroblast, immune, and cancer cells) based on which the dynamic model features ERK induced mRNA and protein expression of DUSP4/16 that catalyze the dephosphorylation of JNK.

#### 2.1.4 AKT inhibits JNK signaling

In response to several growth factors and insulin AKT mediates survival signaling, in part, by phosphorylation and inhibition of apoptotic proteins (Hers et al., 2011). Active AKT phosphorylates inhibitory sites of JNK upstream kinases at both the MAP2K and MAP3K level (see Table 1). On the MAP3K level, phosphorylation of ASK1 at Ser 83 by AKT reduced JNK activity in response to oxidative stress and serum starvation, and decreased ASK1 dependent apoptosis in HEK 293 and L929 cells (Kim et al., 2001). Similar results were obtained in HepG2 cells, where insulin induced AKT activity led to phosphorylation of MLK3 at Ser 674 (Barthwal et al., 2003). On the MAP2K level, AKT phosphorylated MKK4 at Ser 78 in response to insulin or constitutively active AKT, which reduced JNK activity and anisomycin induced apoptosis in HEK 293T cells (Park et al., 2002).

The dynamic model does not distinguish different MAP3Ks and MAP2Ks in the JNK pathway, but features combined ASK/MLK and MKK4/7 components, as MAP3Ks and MAP2Ks respectively. We model both components taking a domain oriented approach (Borisov et al., 2005, 2006; Kiyatkin et al., 2006; Conzelmann et al., 2008) and assuming that the phosphorylation processes at the activation loop and the inhibitory site are independent, as described in detail in *Materials and Methods*.

#### 2.1.5 JNK inhibits ERK and p38

JNK can inhibit ERK on several levels, involving both indirect upstream mechanisms and direct dephosphorylation upon the transcriptional induction of DUSP expression (Junttila et al., 2008). The model of direct ERK dephosphorylation via transcriptional DUSP induction is supported by two studies showing that the JNK-ERK crosstalk is at least partially independent of the ERK upstream kinases MEK and Raf. First, v-Jun transcriptional activity reduced both basal and growth factor induced ERK phosphorylation at least partially independent of Raf (Black et al., 2002). Second, JNK activity induced by ceramide and TNF-α blocked growth factor stimulated ERK phosphorylation, and this inhibition required c-Jun transcriptional activity but did not involve MEK (Shen et al., 2003).

Although the exact mechanism is poorly understood, and elevated expression of DUSP1, DUSP4, and DUSP6 could not be detected in COS-7 cells expressing active MLK3, DUSPs were suggested as potential mediators of the JNK-ERK crosstalk (Shen et al., 2003; Junttila et al., 2008). JNK can also inhibit p38, as JNK activity inhibited both ERK and p38 signaling in mouse cardiomyocytes (Peng et al., 2009) and c-Jun deficient hepatocytes showed increased phosphorylation of p38 (Stepniak et al., 2006). The JNK ⊣ ERK/p38 crosstalk may involve a p53-DUSP2 dependent pathway, as the c-Jun mediated inhibition of p38 observed in hepatocytes was p53 dependent (Stepniak et al., 2006) and DUSP2 was identified as a transcriptional target of p53 in mouse embryonic fibroblast and breast cancer cell lines (Yin et al., 2003). Further, DUSP2 was shown to dephosphorylate ERK and p38 in NIH3T3 and HeLa cells (Chu et al., 1996) and was implicated in the inactivation of ERK2 during p53 dependent apoptosis in breast and colon cancer cell lines (Yin et al., 2003; Dickinson and Keyse, 2006). Based on these data and neglecting p53 as possible intermediate, the dynamic model features JNK induced expression of DUSP2 mRNA and protein and DUSP2-catalyzed dephosphorylation of ERK and p38.

### 2.2 Dynamics of the core network

Based on the model structure in Figure 1 a dynamic model of MAPK interactions can be derived (Kholodenko, 2006; Kholodenko et al., 2010). For detailed introductions to dynamic modeling of cellular systems we refer to (Aldridge et al., 2006; Iglesias and Ingalls, 2009) and, in particular with regards to ERK/MAPK signaling (Kholodenko, 2000; Kolch et al., 2005; Orton et al., 2005). A successful modeling strategy keeps the model simple, yet biologically relevant and capable of meaningful predictions. To that end, the developed model contains several biologically reasonable assumptions, simplifications, and generalizations as explained in *Materials and Methods* (Sec 5). In particular, the model lumps isoforms and kinases that share the same upstream activators and downstream substrates into a single component wherever possible (Figure 1, Tables 4 and 5). Crucially, the adopted simplifications preserve the network’s feedback and crosstalk structures, reduce the risk of over-parameterization and facilitate the mathematical analysis of the model.

#### 2.2.1 MAPK dynamics in response to growth factors or stress

**Figure 2:**
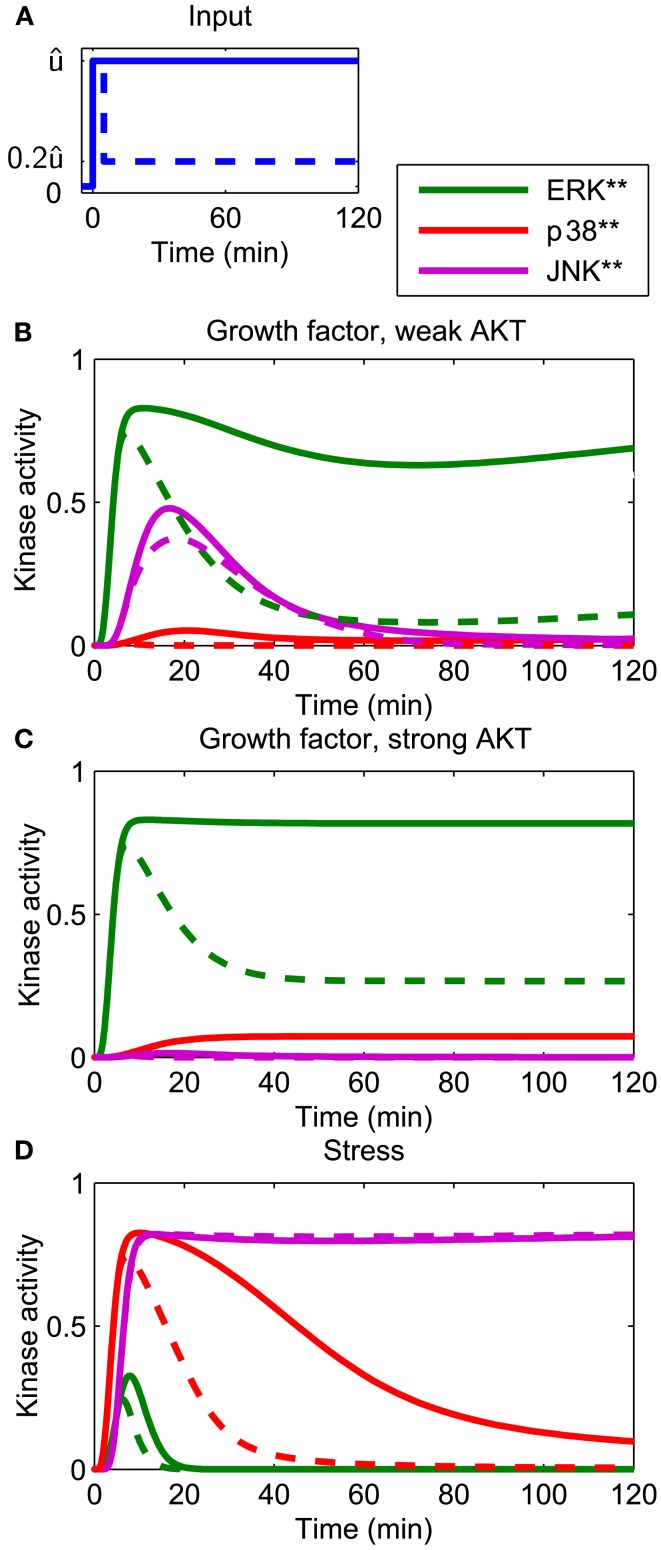
**Dynamics of the core network in response to different stimuli**. **(A)** Form of input signals. Solid: sustained input, dashed: transient input. **(B–D)** System responses for different stimuli. **(B)** Growth factor strongly stimulating the ERK and JNK inputs, but only weakly the AKT and p38 inputs: **(C)** growth factor strongly stimulating the ERK and JNK and AKT inputs, but not the p38 input. **(D)** Stress signal strongly stimulating the p38 and JNK inputs, weakly stimulating the ERK input and not stimulating the AKT input. **Table:** 

The developed model reflects the current understanding of how the p38 and JNK systems respond to stress (Junttila et al., 2008), and is consistent with earlier MAPK models in the literature, which, albeit not concerned with p38 and JNK, featured growth factor induced ERK signaling (von Kriegsheim et al., 2009; Kholodenko et al., 2010; Nakakuki et al., 2010). Figure 2 presents an overview of the system dynamics, illustrating how our model responds to growth factor and stress signals. Generally speaking, growth factors predominantly activate ERK and JNK, and also AKT, albeit to different extents. The activation dynamics may be sustained or transient, depending on type and context of the stimulation (von Kriegsheim et al., 2009; Nakakuki et al., 2010). For example, PC12 cells exhibit sustained ERK activation in response to NGF, whereas EGF causes transient ERK dynamics due to the activation of several negative feedback loops (Marshall, 1995; Douville and Downward, 1997; von Kriegsheim et al., 2009). These negative feedbacks act upstream of the ERK cascade, at the level of growth factor receptors and their adaptors, and result in a transient input signal for the MAPK system. We can model these transient effects taking a modular approach in which the inputs are modeled by time-dependent functions (Nakakuki et al., 2010). Hereby, a step input corresponds to a sustained signal, whereas a pulse input, which drops back to the low basal level after a certain, relatively short time period, corresponds to a transient signal. Figures 2A,B show that in response to growth factors, the ERK dynamics qualitatively follow the input signal, whereas JNK responds transiently, and only to growth factors that do not activate AKT. Stress signals predominantly activate p38 and JNK and sometimes ERK, but to a much weaker extent. Figure 2C shows that the JNK response to stress is sustained for both transient and sustained stress inputs.

	${û}_{\text{ERK}}$	${û}_{\text{p}38}$	${û}_{\text{JNK}}$	${û}_{\text{AKT}}$
**(B)**	1.00	0.15	1.00	0.20
**(C)**	1.00	0.15	1.00	0.50
**(D)**	0.50	1.00	1.00	0.00 where ${û}_{i}$ (*i* = ERK, p38, JNK) denotes the maximal value of the ERK, p38, and JNK input [see **(A)**].

#### 2.2.2 Dynamics of stress induced apoptosis in the presence of growth factors

**Figure 3:**
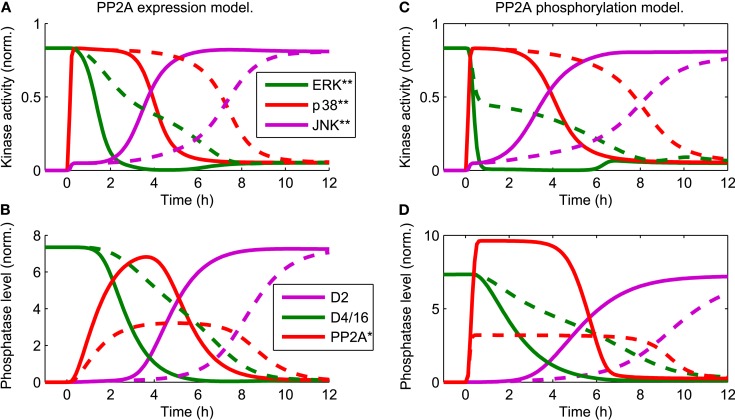
**Trajectories of the core model mimic the sequence of events (Junttila et al., 2008) that occur in response to a stress stimulus *u*_p38_(*t*) = *u*_JNK_(*t*) = 1 for *t* > 0 and the presence of a constant mitotic signal *u*_ERK_(*t*) = 1 for all *t***. D2 and D4/16 denote DUSP2 and DUSP4/16, which mediate the JNK ⊣ ERK, p38, and ERK ⊣ JNK crosstalks, respectively (see also Table 1). The qualitative behavior is independent of the mechanistic details implementing p38-PP2A interaction. Two mechanism are shown: **(A,B)** p38 induces PP2A gene expression, whereby the red line in **(B)** represents the total level of PP2A protein **(C,D)** p38 phosphorylates PP2A, whereby the red line in **(D)** represents phosphorylated PP2A. **(A–D)** The timing of JNK activation depends on the strength of PP2A upregulation: solid lines indicate PP2A levels comparable to those of the other phosphatases. Dashed lines indicate reduced levels of PP2A expression, which delays JNK activation.

The core model reflects the current understanding of JNK dependent apoptosis induction. In Junttila et al. (2008) a conceptual model was proposed, in which PP2A mediated suppression of ERK by p38 is critical for JNK mediated apoptosis. The idea is that stress induced activation of p38 suppresses the normal ERK activity of proliferating and differentiating cells and, subsequently, this loss of ERK activity sensitizes the cells to JNK mediated apoptosis. Our dynamic model is a mathematical representation of this idea amenable to theoretical analysis. Indeed, simulating the dynamic model with a step input of stress signals

$$u_{\text{p}38}\left(t\right)=u_{\text{JNK}}\left(t\right)=\left\{\;\begin{array}{cc} 1 & \text{for }t>0\\ 0 & \text{otherwise} \end{array}\right.$$

in the presence of a constant mitotic signal *u*_ERK_(*t*) = 1 mimics the data and sequence of events described in Junttila et al. (2008). Hereby, the qualitative behavior is largely independent of the exact mechanism of PP2A activation. For both mechanisms, either transcriptional upregulation of PP2A or its activation by p38 induced phosphorylation, the JNK switch occurs at a 3–6 h delay following the apoptotic stimulus (Figure 3). The delay is largely determined by the strength of the p38-PP2A crosstalk. Decreasing the expression rate of PP2A in the transcriptional upregulation model or decreasing the catalytic activity of p38 toward PP2A in the model of phosphorylation induced PP2A activation increases the time of JNK activation (Figure 3, dashed lines). In the following, we dissect the MAPK interaction network generating these complex dynamics, by providing a systems level analysis of these interactions.

### 2.3 Analyzing feedback structures

MAPK systems exhibit complex dynamic behavior, depending on the topology of feedbacks and kinetic parameters. Although the parameters are important for the responses observed, the network topology in terms of feedback loops determines what qualitative behaviors are possible (Kholodenko, 2006). Generally speaking, negative feedback can generate (sustained) oscillations, whereas positive feedback can generate bistability. Bistability is thought to be important in cell fate decisions, as it is characterized by hysteresis and can generate irreversible switches (Novak and Tyson, 1993; Xiong and Ferrell, 2003). An example is the caspase system, where positive feedback generates an irreversible switch between two stable steady states; an off-state corresponding to survival and an on-state corresponding to apoptosis (Eissing et al., 2004). As the model features a JNK positive feedback loop, we sought to determine under which conditions the system exhibits bistability.

#### 2.3.1 Positive feedback and bistability of the JNK module

A convenient tool for analyzing bistability is the loop breaking approach (Angeli et al., 2004). Loop breaking is a graphical analysis tool consisting of two steps. First, break the feedback loop and plot the input/output (i/o) relationship in steady state for the open loop system. The resulting curve is called the steady state characteristic of the open loop. Second, close the loop graphically by plotting a straight line through the origin, whereby the slope of the line represents the inverse strength of the feedback. For example, unitary feedback *u *= *y* is represented by a straight line of slope one. The intersection points of the two lines represent the steady states of the closed loop system. In order to assess the stability of the steady states (similarly to nullclines in classical phase plane analysis), two technical prerequisites have to be satisfied; existence of a well-defined i/o characteristic and monotonicity, both of which can be satisfied for simplified MAPK cascades (not exhibiting negative feedback). For details we refer to the original literature (Angeli et al., 2004).

**Figure 4:**
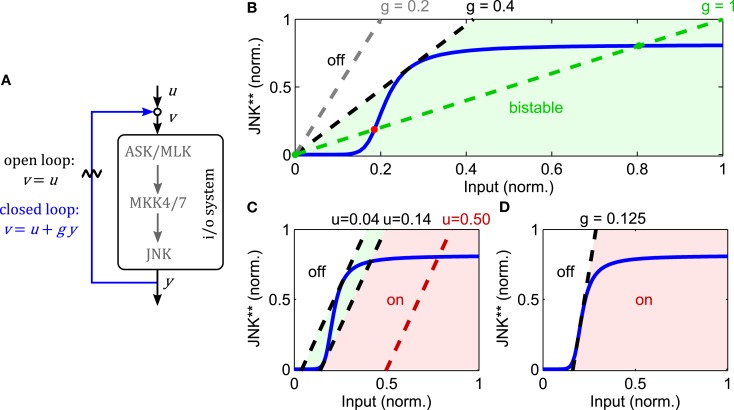
**Analysis of JNK positive feedback using the loop breaking approach**. Here, *g* denotes the feedback strength, i.e., the ratio *g* = *k*_b_/*k*_f_ in (1). **(A–D)** Solid, blue lines represent the steady state characteristic of i/o-system. Dashed lines indicate different feedback configurations, whereby the slope represents the feedback strength and the rightshift measured from the origin the feedforward stimulus. **(A)** Illustration of the loop breaking approach (for a detailed explanation see main text). **(B)** Depending on the feedback strength, the JNK system exhibits monostable or bistable behavior (u = 0). **(C)** Simultaneous feedback and feedforward stimulation can push the system from a monostable-off (white), through a bistable (light green), to a monostable-on (light red) regime. **(D)** No bistable behavior is possible for feedback gains lower than the inverse of the maximal slope of the i/o characteristic.

Zooming into the JNK module of the nominal model, the loop breaking approach reveals that the JNK system is indeed bistable for a wide range of feedback strengths (Figure 4). Note that this result does not depend on the exact parameters, but rather the sigmoidal input-output characteristic of the JNK cascade. In this analysis, the feedback strength corresponds to the catalytic activity of JNK to phosphorylate ASK/MLK. More precisely, let *x*_0_, *x*_1_, and *x*_2_ denote the concentrations of non-, single-, and double-phosphorylated ASK/MLK, accordingly, and let further, *k*_f_ be the catalytic activity of the upstream ASK/MLK input *u* and *k*_b_ the catalytic activity of active JNK *y*, then

(1) $$v_{\text{phos},\;\text{i}}=\frac{\left(k_{\text{f}}û+k_{\text{b}}ŷ\right)x_{i}}{K_{d}+x_{0}+x_{1}},\;i=0,\;1$$

describes the rate of ASK/MLK phosphorylation. Hereby, a feedback strength of 100% corresponds to *k_b_ *= *k_f_*, i.e., equal catalytic activities of input and JNK. Figure 4 shows that for typical values of MAPK phosphorylation and dephosphorylation parameters (Huang and Ferrell, 1996; Kholodenko, 2000; Kholodenko et al., 2010; Nakakuki et al., 2010) the strength of the positive feedback can be reduced to less than 40% before bistability is lost.

The point of transition from monostable to bistable behavior is called pitchfork bifurcation and depends not only on the feedback strength, but also the upstream input. Recall that ASK/MLK are not only phosphorylated by JNK feedback, but also upstream inputs (such as GTPase recruited kinases or MAP4Ks). For the graphical analysis, assuming a constant input corresponds to a rightshift of the feedback line, whereby the value of the rightshift indicates the strength of the input (Figure 4). Applying a feedforward input to a feedback system that was originally not bistable (due to a low feedback gain), can push it into a bistable regime and beyond. Hereby the system moves from a monostable-off regime through a bistable regime to a monostable-on regime (Figure 4). Further, a combined analysis of feedback and feedforward input shows that even for appropriate inputs, bistability is lost if the feedback strength is too low. In fact, in order for a bistable regime to exist, the inverse of the feedback strength has to be smaller than the maximal slope of the sigmoidal i/o characteristic (Figure 4).

#### 2.3.2 Negative feedback via dual specificity kinases

The core model depicted in Figure 1 does not contain negative feedback within the JNK module. However, negative feedback is not uncommon in MAPK cascades and is often context dependent. For instance, ERK possesses several negative feedback loops that are activated in a stimulation dependent manner in response to EGF, but not NGF or HRG (Santos et al., 2007; von Kriegsheim et al., 2009; Nakakuki et al., 2010). With regard to JNK signaling, several DUSPs exhibit catalytic activity toward JNK and may be induced by active JNK (Dickinson and Keyse, 2006; Boutros et al., 2008). One such example is DUSP1 (Bokemeyer et al., 1996). Therefore, we explored the possibility of DUSP1 mediated negative feedback in the JNK module. Note that the system is not monotonic because of the negative feedback. Consequently, graphical analysis using loop breaking cannot assess the stability of the steady states but only their existence and should be complemented by local stability analysis or simulations.

**Figure 5:**
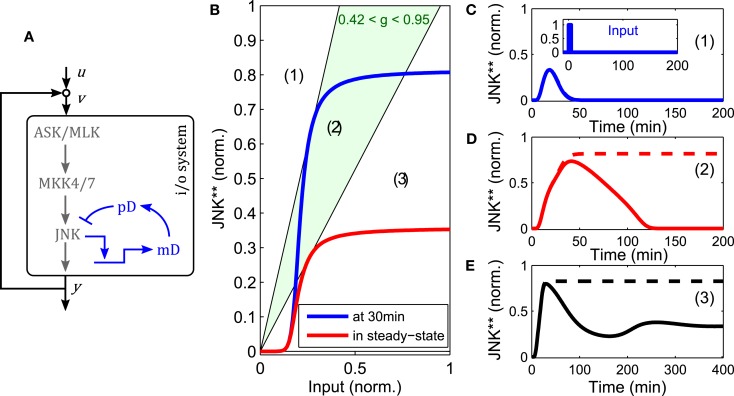
**Modulation of the bistable switch by negative feedback**. **(A)** Scheme of the extended JNK model, in which active JNK induces the expression of DUSP1 mRNA (*mD*) and protein (*pD*). **(B)** Loop breaking analysis showing a transient bistable regime (light green). Blue: initial i/o characteristic of the open loop system at *t* = 30 min, before the negative feedback takes effect. Red: steady state i/o characteristic. **(C–E)** Trajectories of the JNK response after stimulation with a transient pulse of 3 min (*u*(*t*) = 1 for 0 < *t* < 3) for different feedback strengths: **(C)** *g* = 0, **(D)** *g* = 0.7, **(E)** *g* = 1.5. Dashed lines indicate the responses without negative feedback, solid lines with negative feedback.

Negative feedback to upstream components of JNK can decrease ultrasensitivity and lead to oscillations (data not shown, see for example Kholodenko et al., 2010 for a general treatment). In contrast, DUSP1 mediated, slow negative feedback can disable the bistable switch generated by the fast positive feedback loop (Figure 5). Depending on the relative feedback strength, a transiently bistable regime exists, in which the JNK system responds with prolonged activity in response to a short lived stimulus. Hereby, the positive feedback maintains the on-state after the input subsides, but only until the slow negative feedback takes effect, diminishing the (initial) i/o characteristic of the system, at which point JNK switches off (Figure 5).

### 2.4 Regulation of the JNK apoptotic switch by crosstalk

Mitogenic and survival signals regulate the JNK apoptotic switch through crosstalk occurring on several levels (Figure 1). We can distinguish two mechanisms; firstly, inhibition of JNK activation by phosphorylation of upstream JNK kinases at inhibitory residues and secondly enhanced JNK dephosphorylation by upregulation of phosphatases. The first mechanism is mediated by AKT, a classical mediator of survival signaling. The second mechanism, is mediated via ERK, a classical mediator of proliferative and differentiation signaling.

In the following, we use the nominal model to decipher how MAPK crosstalk integrates different mitotic, survival, and stress signals, particularly focusing on the bistable switch. First, we stimulate the model with constant mitotic and survival inputs $u_{\text{ERK}}\left(t\right)={û}_{\text{ERK}},\;u_{\text{AKT}}\left(t\right)={û}_{\text{AKT}}$ and let the trajectories relax to steady state. Then we apply stress stimuli in the form of step inputs

$$\begin{array}{llll} u_{\text{p}38}\left(t\right) & =\left\{\;\begin{array}{cc} 0 & \text{for }t<0\\ {û}_{\text{p}38} & \text{otherwise}, \end{array}\right. & & \\ u_{\text{JNK}}\left(t\right) & =\left\{\;\begin{array}{cc} 0 & \text{for }t<0\\ {û}_{\text{JNK}} & \text{otherwise}. \end{array}\right. & & \end{array}$$

It is useful to define the switching threshold as the value of ${û}_{\text{JNK}}$ at which JNK switches from the off to the on-state.

#### 2.4.1 How AKT controls the JNK switch

**Figure 6:**
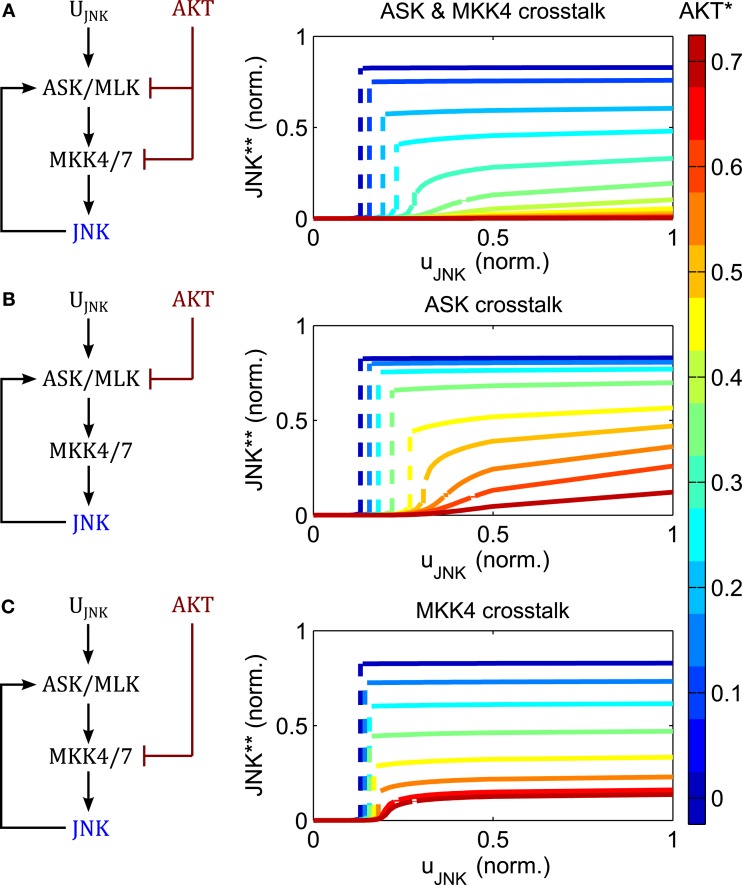
**Regulation of switch by AKT negative crosstalk**. **(A–C)** Interaction schemes and simulated dose responses for crosstalk at different levels: MAP3K and MAP2K level **(A)**; MAP3K level **(B)**; and MAP2K level **(C)**, whereby active AKT phosphorylates and inhibits ASK/MLK and/or MKK4/7 as indicated. Left: interaction scheme. Right: dose responses with respect to the JNK input *u*_JNK_ for different *AKT* activation levels; $u_{\text{JNK}}\left(t\right)={û}_{\text{JNK}}\;\text{for}\;t>0$; $u_{\text{AKT}}={û}_{\text{AKT}}\;\text{for}\;\text{all}\;t$; blue curves indicate low, red lines high AKT activity; dashed lines indicate a switch from low to high JNK activity.

AKT signaling affects the switching threshold and regulates the JNK on-state (Figure 6). Increasing AKT activity decreases the value of the JNK on-state. Whereas bistable behavior is still possible for moderate AKT signaling, strong AKT signaling abrogates the JNK apoptotic switch and permits only moderate, proliferative JNK activity.

The regulation of the JNK switch by AKT does not depend on the exact topology of the crosstalk, as isolated crosstalk at the MAP2K or the MAP3K levels exhibits similar control patterns (Figure 6). One slight difference is that crosstalk on the MAP3K level has slightly more impact on the switching threshold and admits some sensitivity of the proliferative regime with respect to the JNK input, meaning that changing the JNK input changes the level of JNK activity (orange and red curves in Figure 6B). In contrast, the curves resulting from the MAP2K crosstalk are almost flat, meaning that changing the JNK input does not affect JNK activity other than switching it on or off (Figure 6C). Thus, the MAP2K crosstalk model quickly saturates for all levels of AKT activity, after which changing the input has no effect on the output. In contrast, the MAP3K crosstalk model does not saturate when AKT activity is high, and after crossing a certain threshold, JNK responds linearly to changes in the input.

#### 2.4.2 How ERK and p38 control the JNK switch

**Figure 7:**
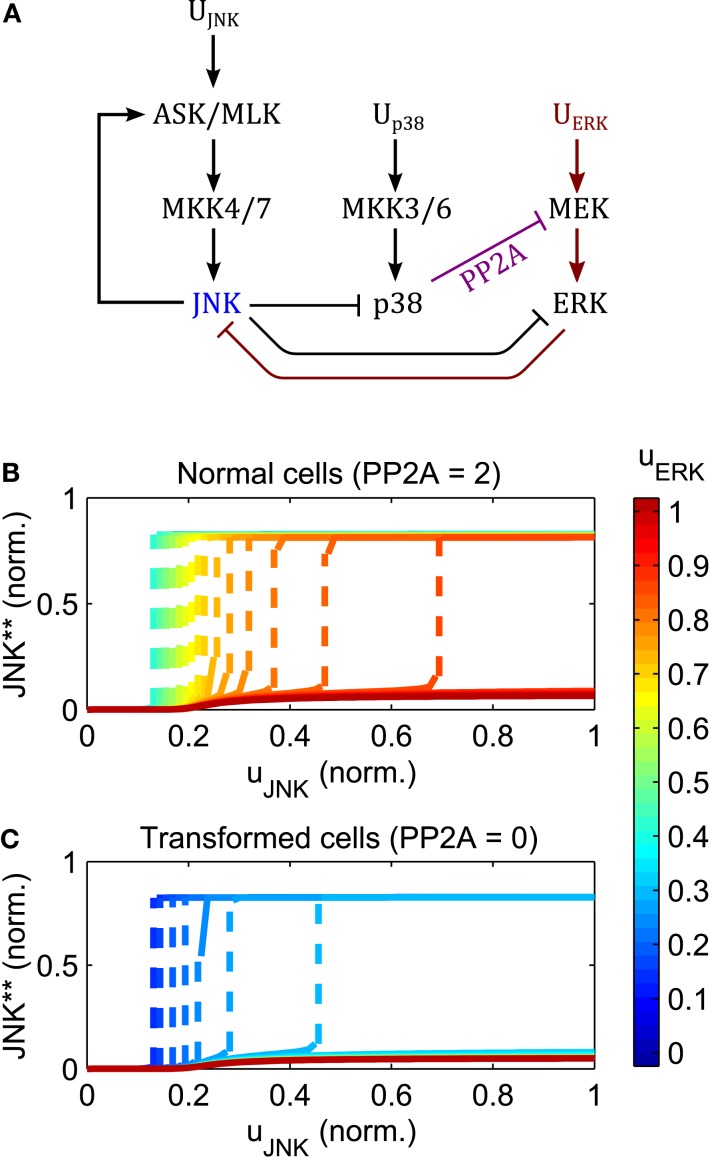
**Regulation of the JNK switch by ERK and p38**. **(A)** Interaction scheme. **(B,C)** Simulated dose responses with respect to the JNK inputs for different levels of constant ERK pathway stimulation; $u_{\text{JNK}}\left(t\right)={û}_{\text{JNK}\;}\text{for}\;t>0$; *u*_p38_(*t*) = 1 for *t* > 0; $u_{\text{ERK}}={û}_{\text{ERK}}\;\text{for}\;\text{all }t$; blue curves indicate low, red lines high ERK stimulation; dashed lines indicate a switch from low to high JNK activity. **(B)** Dose responses for primary/non-transformed cells exhibiting p38 ⊣ ERK crosstalk. **(C)** Dose responses for transformed/tumorigenic cells lacking the p38 ⊣ ERK crosstalk (no PP2A upregulation in the model).

Increasing the input of ERK signaling shifts the switching threshold toward higher JNK inputs, but has little effect on the value of the on-state (Figure 7). Crucially, no intermediate JNK activation is possible, JNK is either off or on, Further, the strength of apoptotic JNK signaling once activated, is independent of the ERK input.

The regulation of the JNK switch by ERK depends on the p38-ERK crosstalk, p38 → PP2A ⊣ ERK. Normal, non-transformed cells can initiate the JNK apoptotic switch depending on the level of PP2A expression and p38 signaling. Here, increasing p38 pathway activation and PP2A expression increases the regime of tolerable ERK stimuli for which JNK inputs can initiate the apoptotic switch (Figure 7B). In contrast, in transformed and tumorigenic cells, lacking p38-ERK crosstalk, even very moderate stimulation of the ERK pathway prevents the JNK apoptotic switch (Figure 7C).

#### 2.4.3 How different DUSP mediated crosstalk patterns shape JNK dynamics

DUSPs are important regulators of MAPK activities. The main function of DUSPs is to dephosphorylate the activation loop of MAPKs, often with overlapping substrate specificity (Dickinson and Keyse, 2006; Boutros et al., 2008; Bermudez et al., 2010). Importantly, several DUSPs are in turn regulated by MAPKs and induced in response to mitotic, differentiation, and stress signals. Therefore, the regulation of DUSPs can occur on several levels including the regulation of DUSP phosphatase activity, substrate specificity, protein stability, and gene expression (Dickinson and Keyse, 2006; Boutros et al., 2008). The resulting feedback and crosstalk structures are complex, and lack a complete understanding. Owing to this complexity, we formulated several models based on reported DUSP specificities in the literature (Table 3; Dickinson and Keyse, 2006; Boutros et al., 2008; Patterson et al., 2009). By focusing on MAPK induced gene transcription and neglecting the complexity of posttranscriptional DUSP regulations, these models are used to analyze the effects of different crosstalk structures.

**Table 3:** **Inducible DUSPs implemented in the model**.

DUSP	Induced by	Substrates	comments/references
DUSP4/16	**ERK** (JNK)	**JNK**, ERK	Oncogenic Ras activity induces DUSP4 mRNA and protein synthesis and stabilizes DUSP4 protein (Cagnol and Rivard, 2012); ERK phosphorylation stabilizes DUSP16 protein, which dephosphorylate JNK ( p38 > ERK, mRNA expression was not analyzed (Katagiri et al., 2005)
DUSP2	**JNK** (ERK)	**ERK**, **p38**	JNK/c-Jun activity and transformed v-JUN enhances ERK dephosphorylation (Black et al., 2002; Shen et al., 2003); DUSP2 was implicated in regulating the JNK ( ERK/p38 crosstalk (Jeffrey et al., 2006); DUSP2 dephosphorylates ERK and p38 (Dickinson and Keyse, 2006)
DUSP1	p38 (ERK, JNK)	p38, JNK	p38 induces DUSP1, which dephosphorylates p38 and JNK (Hu et al., 2007; Small et al., 2007); DUSP1 mRNA is induced by p38 in response to heat shock (marcophages; Wong et al., 2005), anisomycin (VSMC; Bokemeyer et al., 1998), arsenite, UVC (C3H 10T1/2; Li et al., 2001); and by ERK in response to serum (CCL-39; Brondello et al., 1997), PDGF, phorbol ester, angiotensin II (VSMC; Bokemeyer et al., 1998), heat shock, H_2_O_2_ (C3H 10T1/2; Li et al., 2001); and by JNK after stress (NIH 3T3; Bokemeyer et al., 1996)
DUSP5/6	ERK	ERK	MEK/ERK but not PI3K/AKT or p38/JNK regulates DUSP6 mRNA (stabilization) and protein (destabilization) levels (Bermudez et al., 2011) Ras and ERK activity induces dusp5 mRNA and regulates DUSP5 protein stability (Kucharska et al., 2009; Cagnol and Rivard, 2012)

*Bold face indicate activities used in the nominal model. Normal face indicate activities used in Sec. 2.4.3. Round brackets indicate activities not used in this study but implemented in the model, wherewith the model is readily adaptable to cell type and cancer specific interaction patterns by setting the parameters in the model accordingly (see also Table 5)*.

**Table 4:** **Reactions, rate expressions, and parameters of the phosphatase expression processes in the core model**.

Reaction	Forward rate law	Reverse rate law	*K_d_*	*K_−d_*	*k*_1_ (min^−1^)	*k_−_1* (min^−1^)	*k*_2_ (min^−1^)	*k_−_2* (min^−1^)	*k_−_3* (min^−1^)
								
			**unitless**						
$\text{MEK}\overset{{\text{U}}_{\text{ERK}}}{\underset{{\text{PP}2\text{A}}^{*}}{⇌}}{\text{MEK}}^{*}$	$\frac{k_{1}{\text{U}}_{\text{ERK}}\text{MEK}}{K_{d}+\text{MEK}+{\text{MEK}}^{*}}$	$\frac{k_{-1}{\text{PP}2\text{A}}^{*}{\text{MEK}}^{*}}{K_{-d}+{\text{MEK}}^{*}+{\text{MEK}}^{**}}$	1	1	1	0.25	–	–	–
${\text{MEK}}^{*}\overset{{\text{U}}_{\text{ERK}}}{\underset{{\text{PP}2\text{A}}^{*}}{⇌}}{\text{MEK}}^{**}$	$\frac{k_{1}{\text{U}}_{\text{ERK}}{\text{MEK}}^{*}}{K_{d}+\text{MEK}+{\text{MEK}}^{*}}$	$\frac{k_{-1}{\text{PP}2\text{A}}^{*}{\text{MEK}}^{**}}{K_{-d}+{\text{MEK}}^{*}+{\text{MEK}}^{**}}$	1	1	1	0.25	–	–	–
$\text{ERK}\overset{{\text{MEK}}^{**}}{\underset{\text{D}2,\text{D}4∕16,\text{D}5∕6}{⇌}}{\text{ERK}}^{*}$	$\frac{k_{1}{\text{MEK}}^{**}\text{ERK}}{K_{d}+\text{ERK}+{\text{ERK}}^{*}}$	$\frac{\left(k_{-1}\text{D}2+k_{-2}\text{D}4∕16+k_{-3}\text{D}5∕6\right){\text{ERK}}^{*}}{K_{-d}+{\text{ERK}}^{*}+{\text{ERK}}^{**}}$	1	1	1	0.25	–	0	0
${\text{ERK}}^{*}\overset{{\text{U}}_{\text{ERK}}}{\underset{\text{D}2,\text{D}4∕16,\text{D}5∕6}{⇌}}{\text{ERK}}^{**}$	$\frac{k_{1}{\text{U}}_{\text{ERK}}{\text{ERK}}^{*}}{K_{d}+\text{ERK}+{\text{ERK}}^{*}}$	$\frac{\left(k_{-1}\text{D}2+k_{-2}\text{D}4∕16+k_{-3}\text{D}5∕6\right){\text{ERK}}^{**}}{K_{-d}+{\text{ERK}}^{*}+{\text{ERK}}^{**}}$	1	1	1	0.25	–	0	0
$\text{MKK}3∕6\overset{{\text{U}}_{\text{p}38}}{⇌}{\text{MKK}3∕6}^{*}$	$\frac{k_{1}{\text{U}}_{\text{p}38}\text{MKK}3∕6}{K_{d}+\text{MKK}3∕6+{\text{MKK}3∕6}^{*}}$	$\frac{k_{-1}{\text{MKK}3∕6}^{*}}{K_{-d}+{\text{MKK}3∕6}^{*}+{\text{MKK}3∕6}^{**}}$	1	1	1	0.25	–	–	–
${\text{MKK}3∕6}^{*}\overset{{\text{U}}_{\text{p}38}}{⇌}{\text{MKK}3∕6}^{**}$	$\frac{k_{1}{\text{U}}_{\text{p}38}{\text{MKK}3∕6}^{*}}{K_{d}+\text{MKK}3∕6+{\text{MKK}3∕6}^{*}}$	$\frac{k_{-1}{\text{MKK}3∕6}^{**}}{K_{-d}+{\text{MKK}3∕6}^{*}+{\text{MKK}3∕6}^{**}}$	1	1	1	0.25	–	–	–
$\text{p}38\overset{{\text{MKK}3∕6}^{**}}{\underset{\left[\text{D}1,\text{D}2\right]}{⇌}}{\text{p}38}^{*}$	$\frac{k_{1}{\text{MKK}3∕6}^{**}\text{p}38}{K_{d}+\text{p}38+{\text{p}38}^{*}}$	$\frac{\left(k_{-1}\text{D}1+k_{-2}\text{D}2\right){\text{p}38}^{*}}{K_{-d}+{\text{p}38}^{*}+{\text{p}38}^{**}}$	1	1	1	0	–	0.25	–
${\text{p}38}^{*}\overset{{\text{MKK}3∕6}^{**}}{\underset{\text{D}1,\text{D}2}{⇌}}{\text{p}38}^{**}$	$\frac{k_{1}{\text{MKK}3∕6}^{**}{\text{p}38}^{*}}{K_{d}+\text{p}38+{\text{p}38}^{*}}$	$\frac{\left(k_{-1}\text{D}1+k_{-2}\text{D}2\right){\text{p}38}^{**}}{K_{-d}+{\text{p}38}^{*}+{\text{p}38}^{**}}$	1	1	1	0	–	0.2	–
$\text{ASK}\overset{{\text{U}}_{\text{JNK}}}{⇌}{\text{ASK}}^{*}$	$\frac{\left(k_{1}{\text{U}}_{\text{JNK}}+k_{-2}{\text{JNK}}^{**}\right)\text{ASK}}{K_{d}+\text{ASK}+{\text{ASK}}^{*}+{\text{ASK}}_{I}+{\text{ASK}}_{I}^{*}}$	$\frac{k_{-1}{\text{ASK}}^{*}}{K_{-d}+{\text{ASK}}^{*}+{\text{ASK}}^{**}+{\text{ASK}}_{I}^{*}+{\text{ASK}}_{I}^{**}}$	1	1	1	0.25	1	–	–
${\text{ASK}}^{*}\overset{{\text{U}}_{\text{JNK}}}{⇌}{\text{ASK}}^{**}$	$\frac{\left(k_{1}{\text{U}}_{\text{JNK}}+k_{-2}{\text{JNK}}^{**}\right){\text{ASK}}^{*}}{K_{d}+\text{ASK}+{\text{ASK}}^{*}+{\text{ASK}}_{I}+{\text{ASK}}_{I}^{*}}$	$\frac{k_{-1}{\text{ASK}}^{**}}{K_{-d}+{\text{ASK}}^{*}+{\text{ASK}}^{**}+{\text{ASK}}_{I}^{*}+{\text{ASK}}_{I}^{**}}$	1	1	1	0.25	1	–	–
${\text{ASK}}_{I}\overset{{\text{U}}_{\text{JNK}}}{⇌}{\text{ASK}}_{I}^{*}$	$\frac{\left(k_{1}{\text{U}}_{\text{JNK}}+k_{-2}{\text{JNK}}^{**}\right){\text{ASK}}_{I}}{K_{d}+\text{ASK}+{\text{ASK}}^{*}+{\text{ASK}}_{I}+{\text{ASK}}_{I}^{*}}$	$\frac{k_{-1}{\text{ASK}}_{I}^{*}}{K_{-d}+{\text{ASK}}^{*}+{\text{ASK}}^{**}+{\text{ASK}}_{I}^{*}+{\text{ASK}}_{I}^{**}}$	1	1	1	0.25	1	–	–
${\text{ASK}}_{I}^{*}\overset{{\text{U}}_{\text{JNK}}}{⇌}{\text{ASK}}_{I}^{**}$	$\frac{\left(k_{1}{\text{U}}_{\text{JNK}}+k_{-2}{\text{JNK}}^{**}\right){\text{ASK}}_{I}^{*}}{K_{d}+\text{ASK}+{\text{ASK}}^{*}+{\text{ASK}}_{I}+{\text{ASK}}_{I}^{*}}$	$\frac{k_{-1}{\text{ASK}}_{I}^{**}}{K_{-d}+{\text{ASK}}^{*}+{\text{ASK}}^{**}+{\text{ASK}}_{I}^{*}+{\text{ASK}}_{I}^{**}}$	1	1	1	0.25	1	–	–
$\text{ASK}\overset{{\text{AKT}}^{*}}{⇌}{\text{ASK}}_{I}$	$\frac{k_{1}{\text{AKT}}^{*}\text{ASK}}{K_{d}+\text{ASK}+{\text{ASK}}^{*}+{\text{ASK}}^{**}}$	$\frac{k_{-1}{\text{ASK}}_{I}}{K_{-d}+{\text{ASK}}_{I}+{\text{ASK}}_{I}^{*}+{\text{ASK}}_{I}^{**}}$	1	1	1	0.25	–	–	–
${\text{ASK}}^{*}\overset{{\text{AKT}}^{*}}{⇌}{\text{ASK}}_{I}^{*}$	$\frac{k_{1}{\text{AKT}}^{*}{\text{ASK}}^{*}}{K_{d}+\text{ASK}+{\text{ASK}}^{*}+{\text{ASK}}^{**}}$	$\frac{k_{-1}{\text{ASK}}_{I}^{*}}{K_{-d}+{\text{ASK}}_{I}+{\text{ASK}}_{I}^{*}+{\text{ASK}}_{I}^{**}}$	1	1	1	0.25	–	–	–
${\text{ASK}}^{**}\overset{{\text{AKT}}^{*}}{⇌}{\text{ASK}}_{I}^{**}$	$\frac{k_{1}{\text{AKT}}^{*}{\text{ASK}}^{**}}{K_{d}+\text{ASK}+{\text{ASK}}^{*}+{\text{ASK}}^{**}}$	$\frac{k_{-1}{\text{ASK}}_{I}^{**}}{K_{-d}+{\text{ASK}}_{I}+{\text{ASK}}_{I}^{*}+{\text{ASK}}_{I}^{**}}$	1	1	1	0.25	–	–	–
$\text{MKK}4∕6\overset{{\text{ASK}}^{**}}{⇌}{\text{MKK}4∕6}^{*}$	$\frac{k_{1}{\text{ASK}}^{**}\text{MKK}4∕6}{K_{d}+\text{MKK}4∕6+{\text{MKK}4∕6}^{*}+{\text{MKK}4∕6}_{I}+{\text{MKK}4∕6}_{I}^{*}}$	$\frac{k_{-1}{\text{MKK}4∕6}^{*}}{K_{-d}+{\text{MKK}4∕6}^{*}+{\text{MKK}4∕6}^{**}+{\text{MKK}4∕6}_{I}^{*}+{\text{MKK}4∕6}_{I}^{**}}$	1	1	1	0.25	–	–	–
${\text{MKK}4∕6}^{*}\overset{{\text{ASK}}^{**}}{⇌}{\text{MKK}4∕6}^{**}$	$\frac{k_{1}{\text{ASK}}^{**}{\text{MKK}4∕6}^{*}}{K_{d}+\text{MKK}4∕6+{\text{MKK}4∕6}^{*}+{\text{MKK}4∕6}_{I}+{\text{MKK}4∕6}_{I}^{*}}$	$\frac{k_{-1}{\text{MKK}4∕6}^{**}}{K_{-d}+{\text{MKK}4∕6}^{*}+{\text{MKK}4∕6}^{**}+{\text{MKK}4∕6}_{I}^{*}+{\text{MKK}4∕6}_{I}^{**}}$	1	1	1	0.25	–	–	–
${\text{MKK}4∕6}_{I}\overset{{\text{ASK}}^{**}}{⇌}{\text{MKK}4∕6}_{I}^{*}$	$\frac{k_{1}{\text{ASK}}^{**}{\text{MKK}4∕6}_{I}}{K_{d}+\text{MKK}4∕6+{\text{MKK}4∕6}^{*}+{\text{MKK}4∕6}_{I}+{\text{MKK}4∕6}_{I}^{*}}$	$\frac{k_{-1}{\text{MKK}4∕6}_{I}^{*}}{K_{-d}+{\text{MKK}4∕6}^{*}+{\text{MKK}4∕6}^{**}+{\text{MKK}4∕6}_{I}^{*}+{\text{MKK}4∕6}_{I}^{**}}$	1	1	1	0.25	–	–	–
${\text{MKK}4∕6}_{I}^{*}\overset{{\text{ASK}}^{**}}{⇌}{\text{MKK}4∕6}_{I}^{**}$	$\frac{k_{1}{\text{ASK}}^{**}{\text{MKK}4∕6}_{I}^{*}}{K_{d}+\text{MKK}4∕6+{\text{MKK}4∕6}^{*}+{\text{MKK}4∕6}_{I}+{\text{MKK}4∕6}_{I}^{*}}$	$\frac{k_{-1}{\text{MKK}4∕6}_{I}^{**}}{K_{-d}+{\text{MKK}4∕6}^{*}+{\text{MKK}4∕6}^{**}+{\text{MKK}4∕6}_{I}^{*}+{\text{MKK}4∕6}_{I}^{**}}$	1	1	1	0.25	–	–	–
$\text{MKK}4∕6\overset{{\text{AKT}}^{*}}{⇌}{\text{MKK}4∕6}_{I}$	$\frac{k_{1}{\text{AKT}}^{*}\text{MKK}4∕6}{K_{d}+\text{MKK}4∕6+{\text{MKK}4∕6}^{*}+{\text{MKK}4∕6}^{**}}$	$\frac{k_{-1}{\text{MKK}4∕6}_{I}}{K_{-d}+{\text{MKK}4∕6}_{I}+{\text{MKK}4∕6}_{I}^{*}+{\text{MKK}4∕6}_{I}^{**}}$	1	1	1	0.25	–	–	–
${\text{MKK}4∕6}^{*}\overset{{\text{AKT}}^{*}}{⇌}{\text{MKK}4∕6}_{I}^{*}$	$\frac{k_{1}{\text{AKT}}^{*}{\text{MKK}4∕6}^{*}}{K_{d}+\text{MKK}4∕6+{\text{MKK}4∕6}^{*}+{\text{MKK}4∕6}^{**}}$	$\frac{k_{-1}{\text{MKK}4∕6}_{I}^{*}}{K_{-d}+{\text{MKK}4∕6}_{I}+{\text{MKK}4∕6}_{I}^{*}+{\text{MKK}4∕6}_{I}^{**}}$	1	1	1	0.25	–	–	–
${\text{MKK}4∕6}^{**}\overset{{\text{AKT}}^{*}}{⇌}{\text{MKK}4∕6}_{I}^{**}$	$\frac{k_{1}{\text{AKT}}^{*}{\text{MKK}4∕6}^{**}}{K_{d}+\text{MKK}4∕6+{\text{MKK}4∕6}^{*}+{\text{MKK}4∕6}^{**}}$	$\frac{k_{-1}{\text{MKK}4∕6}_{I}^{**}}{K_{-d}+{\text{MKK}4∕6}_{I}+{\text{MKK}4∕6}_{I}^{*}+{\text{MKK}4∕6}_{I}^{**}}$	1	1	1	0.25	–	–	–
$\text{JNK}\overset{{\text{MKK}4∕6}^{**}}{\underset{\text{D}1,\text{D}4∕16}{⇌}}{\text{JNK}}^{*}$	$\frac{k_{1}{\text{MKK}4∕6}^{**}\text{JNK}}{K_{d}+\text{JNK}+{\text{JNK}}^{*}}$	$\frac{\left(k_{-1}\text{D}1+k_{-2}\text{D}4∕16\right){\text{JNK}}^{*}}{K_{-d}+{\text{JNK}}^{*}+{\text{JNK}}^{**}}$	1	1	1	0	–	0.25	–
${\text{JNK}}^{*}\overset{{\text{MKK}4∕6}^{**}}{\underset{\text{D}1,\text{D}4∕16}{⇌}}{\text{JNK}}^{**}$	$\frac{k_{1}{\text{MKK}4∕6}^{**}{\text{JNK}}^{*}}{K_{d}+\text{JNK}+{\text{JNK}}^{*}}$	$\frac{\left(k_{-1}\text{D}1+k_{-2}\text{D}4∕16\right){\text{JNK}}^{**}}{K_{-d}+{\text{JNK}}^{*}+{\text{JNK}}^{**}}$	1	1	1	0	–	0.25	–
$\text{AKT}\overset{{\text{U}}_{\text{AKT}}}{⇌}{\text{AKT}}^{*}$	$\frac{k_{1}{\text{U}}_{\text{AKT}}\text{AKT}}{K_{d}+\text{AKT}}$	$\frac{k_{-1}{\text{AKT}}^{*}}{K_{-d}+{\text{AKT}}^{*}}$	1	1	1	0.25	–	–	–
$\text{PP}2\text{A}\overset{{\text{p}38}^{**}}{⇌}{\text{PP}2\text{A}}^{*}$	$\frac{k_{1}{\text{p}38}^{**}\text{PP}2\text{A}}{K_{d}+\text{PP}2\text{A}}$	$\frac{k_{-1}{\text{PP}2\text{A}}^{*}}{K_{-d}+{\text{PP}2\text{A}}^{*}}$	1	1	1	0.25	–	–	–

*Units: unitless, min. The parameters, and are structural parameters that determine which MAPK induces which phosphatase. The values given in the table relate to the core model*.

**Table 5:** **Reactions, rate expressions and parameters of the phosphatase expression processes in the core model**.

Reaction	Forward rate law	Reverse rate law	*K* (unitless)	*k*_synt_ (min^−1^)	*k*_deg_ (min^−1^)	α	β	γ
						
						**(unitless)**		
$\emptyset \overset{\text{p}38,\text{JNK}}{⇌}\text{dusp}1$	$k_{\text{synt}}\frac{{\left(\alpha \text{p}38+\beta \text{JNK}\right)}^{2}}{K^{2}+{\left(\alpha \text{p}38+\beta \text{JNK}\right)}^{2}}$	*k*_deg_dusp1	0.5	0.0231	0.0231	1	0	0
$\emptyset \overset{\text{dusp}1}{⇌}\text{DUSP}1$	*k*_synt_dusp1	*k*_deg_DUSP1	–	0.231	0.0231	–	–	–
$\emptyset \overset{\text{ERK},\text{JNK}}{⇌}\text{dusp}2$	$k_{\text{synt}}\frac{{\left(\alpha \text{ERK}+\beta \text{JNK}\right)}^{2}}{K_{d}^{2}+{\left(\alpha \text{ERK}+\beta \text{JNK}\right)}^{2}}$	*k*_deg_dusp2	0.5	0.0231	0.0231	0	1	0
$\emptyset \overset{\text{dusp}2}{⇌}\text{DUSP}2$	*k*_synt_dusp1	*k*_deg_DUSP1	–	0.231	0.0231	–	–	–
$\emptyset \overset{\text{ERK},\text{JNK}}{⇌}\text{dusp}4∕16$	$k_{\text{synt}}\frac{{\left(\alpha \text{ERK}+\beta \text{JNK}\right)}^{2}}{K_{d}^{2}+{\left(\alpha \text{ERK}+\beta \text{JNK}\right)}^{2}}$	*k*_deg_dusp4/16	0.5	0.0231	0.0231	1	0	0
$\emptyset \overset{\text{dusp}4∕16}{⇌}\text{DUSP}4∕16$	*k*_synt_dusp4/16	*k*_deg_DUSP4/16	–	0.231	0.0231	–	–	–
$\emptyset \overset{\text{ERK}}{⇌}\text{dusp}5∕6$	$k_{\text{synt}}\frac{{\left(\alpha \text{ERK}\right)}^{2}}{K_{d}^{2}+{\left(\alpha \text{ERK}\right)}^{2}}$	*k*_deg_dusp5/6	0.5	0.0231	0.0231	0	–	0
$\emptyset \overset{\text{dusp}5∕6}{⇌}\text{DUSP}5∕6$	*k*_synt_dusp5/6	*k*_deg_DUSP5/6	–	0.231	0.0231	–	–	–
$\emptyset \overset{{\text{p}38}^{**}}{⇌}\text{pp}2\text{a}$	$k_{\text{synt}}\frac{{\left(\alpha \text{p}38\right)}^{2}}{K_{d}^{2}+{\left(\alpha \text{p}38\right)}^{2}}$	*k*_deg_pp2a	0.5	0.0231	0.0231	0	–	0
$\emptyset \overset{\text{pp}2\text{a}}{⇌}\text{PP}2\text{A}$	*k*_synt_pp2a	*k*_deg_PP2A	–	0.231	0.0231	–	–	–

*The parameters α, β, and γ are structural parameters ∈{0, 1} that determine which MAPK induces which phosphatase. The values given in the table relate to the core model*.

DUSP1 expression can be induced by active p38 and JNK depending on the cell context (Table 3), and it is often upregulated in cancer. The JNK induced DUSP1 expression and the resulting negative feedback onto JNK was already analyzed in Sec. 2.3.2, Figure 5. In this section we focus on the p38 induced DUSP1 expression and resulting p38 ⊣ p38/JNK crosstalk. We have already seen in Sec. 2.4.2, Figure 7 that p38 ⊣ ERK ⊣ JNK crosstalk is a critical regulator of the JNK switch. However, the core model also features JNK ⊣ ERK/p38 crosstalk mediated by DUSP2 expression, and we ask whether this crosstalk is also crucial for the JNK switch by deleting DUSP2 in the model.

**Figure 8:**
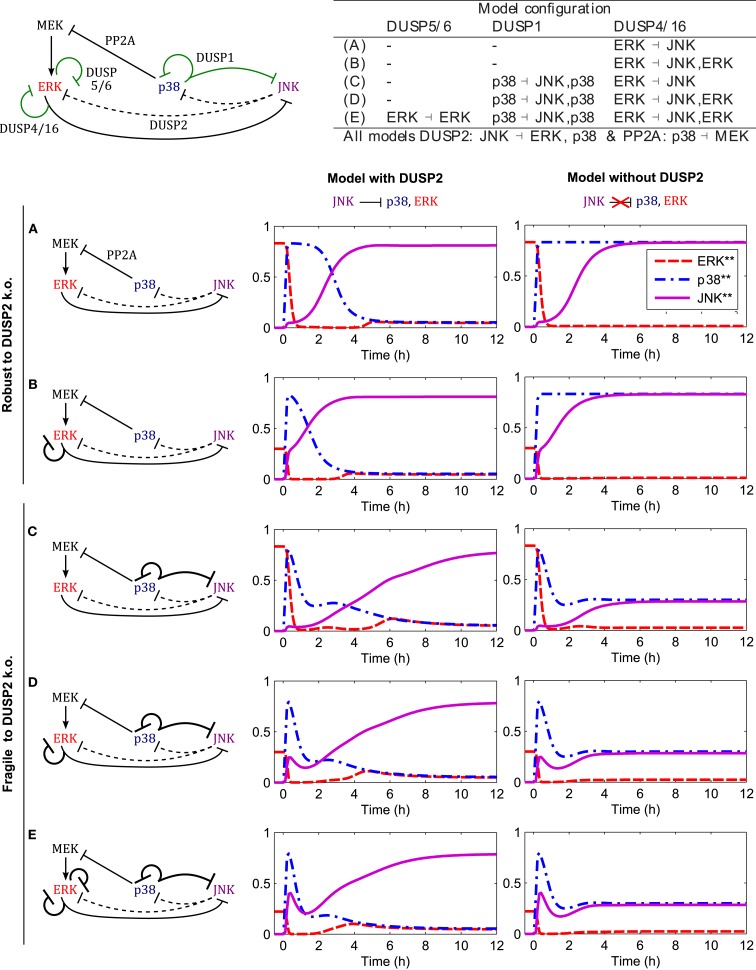
**Dynamics of the apoptotic switch for different crosstalk patterns**. Center and right columns show the trajectories of MAPKs activation after a stress stimulus (*u*_p38_ = *u*_JNK_ = 1 for *t* > 0) in the presence of a mitotic signal (*u*_ERK_ = 1 for all *t*) for the indicated interaction patterns. Top left: scheme of MAPK interactions. Black arcs indicate core interactions, green arcs indicate additional DUSP mediated interactions analyzed in panels **(A–E)**. Top right: table summarizing the different DUSP interaction patterns corresponding to **(A–E)**. **(A)** Core model (see Figure 1 for a detailed scheme). **(B)** Core model and DUSP4 mediated negative feedback on ERK. **(C)** Core model and p38 induced DUSP1 expression mediating negative feedback to ERK and crosstalk to JNK: p38 ( p38/JNK. **(D)** Model C and DUSP4 mediated negative feedback on ERK. **(E)** Model D and DUSP5/6 mediated negative feedback on ERK.

Figure 8 shows the responses for c patterns to step inputs of stress signals,

$$u_{\text{p}38}\left(t\right)=u_{\text{JNK}}\left(t\right)=\left\{\;\begin{array}{cc} 1 & \text{for }t>0\\ 0 & \text{otherwise} \end{array}\right.$$

in the presence of a constant mitotic signal *u*_ERK_(*t*) = 1 for all *t*. We can distinguish two qualitatively different behaviors, irrespective of the presence or absence of ERK feedback (mediated by DUSP4 or DUSP5/6). The models in the first group do not feature p38 induced DUSP1 expression, and DUSP2 deletion in these models has little effect on the JNK activation dynamics and the JNK switch (Figures 8A,B). Within this group, model A is the core model, but model B also includes DUSP4 mediated negative feedback to ERK; ERK ⊣ ERK/JNK (Table 3), resulting in accelerated JNK activation dynamics (Figure 8B). In contrast, the models in the second group feature p38 induced DUSP1 expression, and deletion of DUSP2 in these models abrogates the JNK switch, resulting in reduced, moderate JNK activity (Figures 8C–E). In addition to the core interactions, model C includes this p38 induced DUSP1 expression, which slightly delays the JNK activation dynamics, but does not obliterate the JNK switch (Figure 8C). However, deleting DUSP2 in model C abrogates the JNK switch and results in only moderate JNK activity (Figure 8C). Summarizing, these models predict that abrogation of JNK dependent apoptosis requires both p38 induced expression of DUSP1 and downregulation or deletion of DUSP2. Adding ERK negative feedback mediated by DUSP4 (model D) or DUSP4 and DUSP5/6 (model E) to model C does not alter the JNK dynamics or the behavior of the DUSP2 deletion (Figures 8D,E).

The robustness of the core model with respect to either (i) gain of p38 induced DUSP1 or (ii) loss of JNK induced DUSP2 in isolation, can be explained as follows. To lock JNK into the highly active state (in the presence of ERK input), ERK activity needs to be suppressed, either by p38 activity via the PP2A-ERK link or by JNK activity via the DUSP2-ERK link. In the absence of DUSP1, p38 activity is sufficiently high to suppress ERK. In presence of DUSP1, the p38 activity is reduced, and ERK is not sufficiently suppressed by p38-PP2A alone. Here, the JNK-DUSP2-ERK crosstalk becomes crucial as it complements the p38-PP2A mediated ERK inhibition, which explains the fragility of the JNK switch if both elevation of p38 induced DUSP1 and loss of JNK induced DUSP2 occur simultaneously.

## 3 Discussion

The process of building a multi-pathway model is quite complex, as are the implications of its analysis for cell biology and cancer. Both are discussed in the following.

### 3.1 Theoretical considerations

The network depicted in Figure 1 synthesizes information from different cell types in the literature. However, a complete picture of MAPK crosstalk is still lacking. The network of DUSP interactions is particularly difficult to dissect, as DUSPs can be induced by several MAPKs and, in turn, can act on several substrates (Boutros et al., 2008). Importantly, Figure 1 is not an overview summarizing all possible interactions, but depicts a core model of MAPK interactions that are essential for implementing the JNK proliferative-apoptotic switch. From a systems-theoretical perspective, the crucial determinant of the model’s behavior is that these crosstalks and feedbacks exists, not which molecules mediate it. Therefore, although all interactions in the model are strongly supported by experimental evidence in the literature, the particular DUSP isoforms involved may differ depending on cell type and context. The DUSP2 connection in our model is based on data from fibroblast and cancer cell lines (see Sec. 2.1.5), but is particularly uncertain. DUSP2 specifically dephosphorylated ERK and p38 in NIH3T3 and HeLa cells (Chu et al., 1996), but targeted JNK in macrophages and mast cells (Jeffrey et al., 2006). These differences suggest that the JNK ⊣ ERK/p38 crosstalk may be mediated differently in immune cells compared to fibroblasts and epithelial cancer cells. In concordance with our model, macrophages, and mast cells exhibited JNK ⊣ ERK/p38 crosstalk, but unlike our model this crosstalk was not mediated by DUSP2, as DUSP2 deletion in macrophages and mast cells increased JNK activity and decreased ERK and p38 phosphorylation (Jeffrey et al., 2006). These cell type specific differences highlight the importance of flexible modeling approaches that facilitate the analysis of different model configurations. As illustrated in Section 2.4.3, testing alternative model topologies can readily be done by setting parameters in the current model.

#### 3.1.1 Modularity and neglected components

The nominal model neglects several context dependent MAPK crosstalks and feedbacks that are not necessary for implementing the JNK apoptotic switch. For example, ERK features several (negative) feedback loops (Birtwistle et al., 2007; von Kriegsheim et al., 2009). Generally, this feedback acts upstream of MEK (on the components that are not included in the model, such as growth factor receptors, their adaptors and GTPases), thereby transiently shaping Raf activation, i.e., the input of the model *u*_1_. Thus, although the model does not account for ERK feedbacks explicitly, it can account for different Raf activation patterns by choosing *u*_1_ accordingly as a time-dependent input function (Nakakuki et al., 2010). One advantage of this modular approach is that it facilitates further model development as the input functions can be replaced by additional sets of differential equations. Therewith, the model can be easily connected to other models describing the dynamics of different receptors and GTPases.

Future model development will concern including more mechanistic detail, in particular with regard to the well-studied ERK cascade. For instance, ERK exhibits a strong negative feedback to Raf-1 in response to EGF which alters the efficiency of MEK inhibition (Sturm et al., 2010) and might affect the p38-PP2A-MEK crosstalk. In contrast, IGF predominantly activates ERK via B-Raf without featuring negative feedback (Fritsche-Guenther et al., 2011), illustrating that these model extensions will be context and stimulus dependent.

#### 3.1.2 Spatial aspects

Mathematically, the model describes the cell as well mixed compartment and does not distinguish subcellular compartments. This simplification might not be an issue for the ERK-JNK crosstalk as it features both a nuclear (DUSP4) and a cytosolic (DUSP16) component, but might overestimate the effects of the JNK-ERK/p38 and p38-JNK crosstalks, because their mediators DUSP2 and DUSP1 are exclusively localized in the nucleus. In general, the spatial regulation of DUSPs and MAPKs is complex, as DUSPs can both shuttle and sequester MAPKs in the nucleus and cytosol (Masuda et al., 2001; Karlsson et al., 2004; Mandl et al., 2005; Caunt and Keyse, 2012). In addition, many MAPKs and their MAP2Ks also shuttle between the cytosol and nucleus, and activation and deactivation can take place in both compartments (Plotnikov et al., 2011). For instance, MKK3/6 are located both in the cytosol and nucleus and can mediate p38 activation in the nucleus (Ben-Levy et al., 1998). Both ERK and MEK constitutively shuttle between the cytosol and nucleus, and ERK can be activated in both compartments (Fujioka et al., 2006). Likewise, ERK deactivation by DUSPs can occur in the nucleus and cytosol depending on the localization of particular DUSP isoforms. Nuclear DUSPs seem to serve as anchoring proteins that retain dephosphorylated ERK in the nucleus to prevent re-activation in the cytosol (Lenormand et al., 1998). Thus, spatial context seems to play an intricate role in modulation of MAPK activities, more work would will be needed to decipher and model the spatial regulations of DUSPs and their effects on MAPK activities.

#### 3.1.3 Parameter dependency

The parameterization of dynamic models is complex, usually requiring time-course measurements in several different conditions and fitting of the model using global optimization algorithms, whereby the resulting parameter estimates might vary depending on cell type and experimental context. To obtain a nominal model, we chose the parameters values in concordance with earlier models of MAPK signaling and kinetic information in the literature, such as half life measurements of DUSPs (see Materials and Methods). For simplification, the model assumed equal parameters for different MAPKs. We do not expect this assumption to withstand experimental validation during parameter estimation as it was adopted for theoretical reasons. (We refer to von Kriegsheim et al., 2009; Cirit et al., 2010; Nakakuki et al., 2010 for compilations of kinetic parameters and Legewie et al., 2008 for turnover rates.) First, assuming equal binding constants for kinases acting on shared substrates established symmetries in the model that yielded simplified, Michaelis-Menten-like kinetic expressions in a model reduction step (see *Materials and Methods*). Second, choosing equal catalytic activities for the different MAPKs simplifies analyzing the model based on the rationale that, in this case, the system dynamics are dominated by the systems structure and not biased toward possible imbalances of particular parameter values. Nonetheless, an important feature of the model is that the bistable nature of the JNK apoptotic switch does not depend on the exact parameter values used, but relies on feedback structure and sigmoidal shape of the i/o characteristic. Nevertheless, in order to achieve a quantitatively predictive model, future work would be needed for data collection and parameterization, particularly with regard to stress activated kinases where little kinetic information is available.

### 3.2 Biological implications

Although the idea of a JNK positive feedback loop is not new (Ventura et al., 2006), a detailed understanding of how JNK feedback structures and crosstalk regulate cell fate is still missing (Wagner and Nebreda, 2009). Earlier modeling studies considered MAPK systems in isolation from each other, either focusing on ERK signaling and its feedbacks (von Kriegsheim et al., 2009; Kholodenko et al., 2010; Nakakuki et al., 2010; Sturm et al., 2010), or p38-JNK crosstalk (Sundaramurthy et al., 2009; Sundaramurthy and Gakkhar, 2010). In contrast, this manuscript provides a systems level model of JNK positive feedback, its regulation by pathway crosstalk including ERK and AKT signaling, and a mathematical analysis of how this system integrates different proliferative, survival, and proapoptotic stimuli, thereby determining cell fates. The model helps us to understand the experimental observations in the literature, and incorporates several ideas. First, the magnitude and temporal profile of JNK signaling is important, as the anti-apoptotic, proliferative response is associated with moderate, but rapid JNK activation, whereas the proapoptotic response is associated with later, more sustained JNK activation (Lamb et al., 2003; Sakon et al., 2003; Ventura et al., 2006). Second, both mitotic signaling via ERK and survival signaling via AKT modulate the JNK apoptotic switch (Molton et al., 2003; Junttila et al., 2008). Third, the loss of negative crosstalk from p38 to ERK dysregulates JNK dependent apoptosis, which is crucial for cell transformation (Arroyo and Hahn, 2005; Junttila et al., 2008). Overall, JNK signaling involving a positive feedback loop takes a center stage in the proposed model, which explains how ERK and AKT mediated crosstalk modulates and switches proliferative and proapoptotic JNK signaling.

#### 3.2.1 Differences of ERK and AKT control over the JNK apoptotic switch

In the model, the switch to apoptotic JNK signaling depends crucially on a JNK positive feedback loop, which, once activated, causes high levels of sustained JNK activity. This switch is modulated by ERK and AKT signaling in different ways. ERK activity shifts the threshold for the JNK apoptotic switch to higher values, but has no effect on the strength of apoptotic JNK signaling. The mechanism underlying this behavior is the enhanced dephosphorylation of JNK, whereby JNK activity is either sufficient to activate the JNK positive feedback loop and inhibit ERK signaling, or does not reach this threshold level. In contrast, AKT activity predominantly regulates the strength of JNK signaling by reducing the value of the JNK on-state with little effect on the switching threshold. The mechanism behind this is the phosphorylation and inhibition of JNK upstream kinases, which reduces the strength of both, the feedforward loop and the feedback loop. Crucially, the reduced feedback strength yields a reduced level of the JNK on-state.

#### 3.2.2 Transformed versus normal cells

Transformed cell differ from normal cells in that they lack PP2A mediated negative crosstalk from p38 to ERK (Junttila et al., 2008). In the model, the loss of p38-ERK negative crosstalk severely increases the JNK switching threshold, thus desensitizing the cells from stress induced apoptosis. Taken together, these observations suggest the involvement of the JNK apoptotic switch in cellular senescence as follows. With each cell cycle, cells accumulate DNA damage and experience a shortening of the chromosomal telomeres. Once a certain threshold of DNA damage or telomere shortening is crossed, senescence occurs, or apoptosis is induced. The loss of p38-ERK crosstalk would increase this threshold to unphysiological levels, thus rendering transformed cells biologically immortal. In this senescence model, AKT is not involved, as the AKT-JNK crosstalk does not alter the apoptotic threshold, but, instead, prevents the apoptotic switch in the presence of survival signals.

## 4 Conclusions

The developed model explains how pathway crosstalk harmonizes MAPK responses resulting in pivotal cell fate decisions that differ markedly between transformed and non-transformed cells. In the proposed model, JNK can switch from a transient to sustained activity due to multiple positive feedback loops. Once activated, positive feedback locks JNK into a highly active state that promotes cell death. The switch is differentially regulated by the ERK, p38, and AKT pathways. ERK activation enhances the dual specify phosphatase (DUSP) mediated dephosphorylation of JNK and shifts the threshold of the apoptotic switch to higher inputs. In non-transformed cells, activation of p38 can restore the threshold by inhibiting ERK activity via the phosphatases PP1 or PP2A. Finally, AKT activation inhibits the JNK positive feedback, thus abrogating the apoptotic switch and allowing only proliferative signaling. The model is most valuable for understanding how cancerous deregulations disturb the signal processing of internal and external cues and provides possible explanations for certain drug resistances. For instance, oncogene induced ERK hyperactivity prevents the normal apoptotic switch and provides possible explanations for the complex and tumor specific behavior of MAPK systems (Dickinson and Keyse, 2006; Wagner and Nebreda, 2009; Bermudez et al., 2010).

In regards to interactions necessary for facilitating the switch between transient and sustained JNK activity, our model predicts a critical role for DUSP1 and DUSP2 expression patterns. In the model, both expression of DUSP1 and deletion of DUSP2 are necessary for preventing the JNK apoptotic switch (as the nominal model is robust to dysregulation of either DUSP in isolation). The result is particularly interesting in the context of (a) cancer, as many cancers show increased expression of DUSP1 and reduced expression of DUSP2, and (b) tumor related conditions such as hypoxia, where low oxygen levels upregulate DUSP1 and downregulate DUSP2 (Patterson et al., 2009; Lin et al., 2011). According to our model, these conditions would prevent the JNK apoptotic switch. Indeed, forced expression of DUSP2 abolished hypoxia induced chemoresistance in human cancer cell lines (Lin et al., 2011), and inhibition of DUSP1 sensitized several resistant cancer cell lines to JNK dependent apoptosis (Laderoute et al., 1999; Sánchez-Pérez et al., 2000; Small et al., 2007; Wang et al., 2008).

The current model represents a core network of MAPK interactions critical for the switch from proliferative to apoptotic signaling. The model is canonical in the sense that it generalizes and integrates information from different cell lines by focusing on interactions that are (a) readily observed in several cell lines and (b) important for controlling the JNK bistable switch. The canonical model forms a basis for experimental design and can be tailored to different experimental systems on two levels by (a) parameter estimation (the epigenetic and disease background of particular cell types is reflected in different parameter values of the model) and (b) extending the model to incorporate different MAPK isoforms, upstream and downstream signaling and scaffolds. Such refined and validated models possess quantitative predictive power and cannot only be used for identifying gaps in knowledge by testing the model predictions, but also for predicting the effect of drugs, thus building the theoretical basis for identifying optimal treatment strategies.

## 5 Materials and Methods

This section describes how the different components depicted in Figure 1 are modeled mathematically.

### 5.1 Model of kinase activation

**Figure 9:**
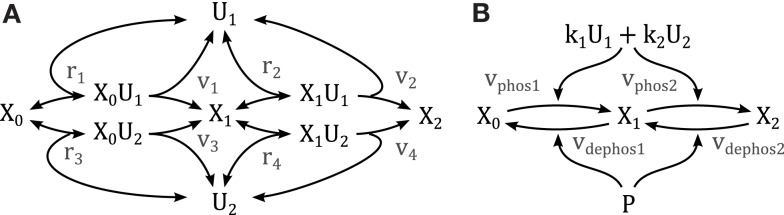
**Schemes of a double phosphorylation cycle with two kinases**. *X* denotes the protein to be phosphorylated with the index indicating its phosphorylation status. *U* denote the kinases catalyzing the phosphorylations. **(A)** Full mechanistic scheme modeled using mass action kinetics. For clarity of presentation, the dephosphorylation reactions are not depicted. **(B)** Reduced scheme modeled using Michaleis-Menten type kinetics as derived in the main text; *k*_1_ and *k*_2_ denote the catalytic activity of *U*_1_ and *U*_2_, respectively. *P* denotes the phosphatase catalyzing the dephosphorylation.

Activation of mitogen-activated protein kinases requires the phosphorylation of two conserved amino acid residues, whereby several upstream kinases can act as enzymes facilitating the phosphorylation. Consider the reaction scheme in Figure 9, where we assumed that only one enzyme can be bound to the kinase at any one time. Using the law of mass action the reaction rates are described by

$$\begin{array}{llll} & r_{1}=p_{1}x_{0}u_{1}-p_{-1}x_{0u1}, & & \text{(2a)}\\ & v_{1}=k_{1}x_{0u1}, & & \text{(2b)}\\ & r_{2}=p_{1}x_{1}u_{1}-p_{-1}x_{1u1}, & & \text{(2c)}\\ & v_{2}=k_{1}x_{1u1}, & & \text{(2d)}\\ & r_{3}=p_{2}x_{0}u_{2}-p_{-2}x_{0u2}, & & \text{(2e)}\\ & v_{3}=k_{2}x_{0u2}, & & \text{(2f)}\\ & r_{4}=p_{2}x_{1}u_{1}-p_{-2}x_{1u2}, & & \text{(2g)}\\ & v_{4}=k_{2}x_{1u2}, & & \text{(2h)} \end{array}$$

and the dynamics of the system are governed by the ordinary differential equations

$$\begin{array}{llll} \frac{dx_{0}}{dt} & =-r_{1}-r_{3}, & & \text{(3a)}\\ \frac{dx_{1}}{dt} & =v_{1}-r_{2}+v_{3}-r_{4}, & & \text{(3b)}\\ \frac{dx_{2}}{dt} & =v_{2}+v_{4}, & & \text{(3c)}\\ \frac{dx_{0u1}}{dt} & =r_{1}-v_{1}, & & \text{(3d)}\\ \frac{dx_{1u1}}{dt} & =r_{2}-v_{2}, & & \text{(3e)}\\ \frac{dx_{0u2}}{dt} & =r_{3}-v_{3}, & & \text{(3f)}\\ \frac{dx_{1u2}}{dt} & =r_{4}-v_{4}. & & \text{(3g)} \end{array}$$

We can obtain a simplified description of this system resembling classical Michaelis Menten kinetics using the conserved moieties for the enzymes

$$u_{i}={û}_{i}-x_{0ui}-x_{1ui},\;\left(i=1,\;2\right),$$

where ${û}_{i}$ denotes the total concentration of enzyme *i*, and assuming rapid equilibrium for the binding reactions by solving the system *r_i_* = 0 (*i* = 1, …, 4) for the complexed states *x*_0*u*1_, *x*_1*u*1_, *x*_0*u*2_, *x*_1*u*2_. Substituting the solution into the phosphorylation reactions *v_i_* (*i* = 1, …, 4) yields

$$\begin{array}{llll} v_{1}=\frac{{û}_{1}k_{1}x_{0}}{K_{d1}+x_{0}+x_{1}}, & & & \text{(4a)}\\ v_{2}=\frac{{û}_{1}k_{1}x_{1}}{K_{d1}+x_{0}+x_{1}}, & & & \text{(4b)}\\ v_{3}=\frac{{û}_{2}k_{2}x_{0}}{K_{d2}+x_{0}+x_{1}}, & & & \text{(4c)}\\ v_{4}=\frac{{û}_{2}k_{2}x_{1}}{K_{d2}+x_{0}+x_{1}}, & & & \text{(4d)} \end{array}$$

with *K_di_* = *p*_−*i*_/*p_i_* (*i* = 1, 2) denoting the dissociation constant of the enzyme-kinase complexes. We can further simplify the model by assuming equal dissociation constants *K_di_* = *K_d_*, wherewith

$$\begin{array}{llll} v_{\text{phos}1} & =v_{1}+v_{3}=\frac{\left(k_{1}{û}_{1}+k_{2}{û}_{2}\right)x_{0}}{K_{d}+x_{0}+x_{1}}, & & \text{(5a)}\\ v_{\text{phos}2} & =v_{2}+v_{4}=\frac{\left(k_{1}{û}_{1}+k_{2}{û}_{2}\right)x_{1}}{K_{d}+x_{0}+x_{1}}. & & \text{(5b)} \end{array}$$

Assuming equal dissociation constants is a strong assumption, in particular for different enzymes, but reduces the risk of over-parameterization and facilitates the theoretical analysis of the model.

Dephosphorylation of kinases is catalyzed by phosphatases and a mathematical model can be derived analogously, resulting in kinetic expressions resembling equation (5). Therewith, a complete model of a double phosphorylation cycle is given by

$$\begin{array}{l} \frac{d}{dt}x_{0}=-\sum_{i}\left(k_{i}{\hat{u}}_{i}\right)\frac{x_{0}}{K_{d}+x_{0}+x_{1}}\\ \;+\sum_{j}\left(k_{-j}{\hat{v}}_{j}\right)\frac{x_{1}}{K_{-d}+x_{1}+x_{2}},\\ \frac{d}{dt}x_{2}=\sum_{i}\left(k_{i}{\hat{u}}_{i}\right)\frac{x_{1}}{K_{d}+x_{0}+x_{1}}\\ \;-\sum_{j}\left(k_{-j}{\hat{v}}_{j}\right)\frac{x_{2}}{K_{-d}+x_{1}+x_{2}}, \end{array}$$

where *u_i_* denote the concentrations of the upstream kinases, *v_j_* the concentrations of the phosphatases.

### 5.2 Model of kinase inhibition by phosphorylation

Some kinases can be rendered catalytically inactive by phosphorylation at inhibitory sites. Examples are ASK1 and MKK4, which can be phosphorylated by AKT at Ser 83 and Ser 78, respectively (Kim et al., 2001; Park et al., 2002). There is little, mostly conflicting information available on whether phosphorylation of the inhibitory site depends on the phosphorylation status of the activating sites, or, in turn, whether phosphorylation at the inhibitory site affects the phosphorylation/dephosphorylation of the activating sites. Hence, we take a domain oriented approach accounting for all possible combinations of the phosphorylation status (but neglecting the possibility of a trimeric complex), resulting in a model comprising six distinct states (Borisov et al., 2005, 2006; Kiyatkin et al., 2006; Conzelmann et al., 2008).

Assume that the phosphorylation status of the activating site does not affect the binding of the inhibitor enzyme and the phosphorylation of the inhibitory site, and vice versa that the phosphorylation status of the inhibitory site does not affect the binding of the activating enzyme and the phosphorylation of the activating site. Assume further, that only one enzyme can be bound to the kinase at any one time, i.e., a trimeric complex consisting of activating enzyme, kinase, and inhibitory enzyme is not possible. Then the reaction scheme in Figure 9 can be extended to account for kinase inhibition:

$$X_{i}\overset{U_{I}}{⇌}X_{iI},\;\left(i=0,1,2\right),$$

where *X_iI_* denotes the kinase phosphorylated at the inhibitory site and *U_i_* the enzyme catalyzing this phosphorylation.

Similarly to equation (5) a mathematical description can be derived, yielding reaction kinetics for (de-)phosphorylation at the activating sites of the form

$$\begin{array}{llll} r_{\text{phos}} & =\frac{\sum_{l}\;k_{l}{û}_{l}y}{K_{d}+x_{0}+x_{1}+x_{0I}+x_{1I}}, & & \\ r_{\text{dephos}} & =\frac{\sum_{l}\;k_{-l}{\hat{v}}_{-l}z}{K_{-d}+x_{1}+x_{2}+x_{1I}+x_{2I}}, & & \end{array}$$

where (*y*, *z*) ∈ {(*x*_0_, *x*_1_), (*x*_1_, *x*_2_), (*x*_0*I*_, *x*_1*I*_), (*x*_0*I*_, *x*_1*I*_)} and reaction kinetics for (de-)phosphorylation of the inhibitory site of the form

$$\begin{array}{llll} r_{\text{phos},\text{I}} & =\frac{k_{I}{û}_{I}y}{K_{I}+x_{0}+x_{1}+x_{2}}, & & \\ r_{\text{dephos},\text{I}} & =\frac{k_{-I}{\hat{v}}_{I}z}{K_{-I}+x_{0I}+x_{1I}+x_{2I}}, & & \end{array}$$

where (*y*, *z*) ∈ {(*x*_0_, *x*_0*I*_), (*x*_1_, *x*_1*I*_), (*x*_2_, *x*_2*I*_)}.

### 5.3 Model of phosphatase expression

The expression levels of inducible phosphatases depend on several parameters, including the activities of upstream MAPKs (Figure 1). We model the rate of mRNA synthesis using sigmoidal Hill functions of activator kinase concentrations (motivated by thermostatistical arguments; Frank et al., 2012) and assume first order kinetics for translation and degradation rates. Therewith a dynamic model of gene expression is described as

$$\begin{array}{llll} \frac{d}{dt}x & =k_{\text{synt}}\frac{u^{n}}{K^{n}+u^{n}}-k_{\text{deg}}x, & & \\ \frac{d}{dt}y & =p_{\text{synt}}x-p_{\text{deg}}y, & & \end{array}$$

where *u* denotes the concentration of upstream kinase activity, *x* and *y* denote mRNA and protein concentrations, respectively, and *k*_synt_, *K*, *n*, *k*_deg_, *p*_synt_, and *p*_deg_ are kinetic parameters. Many of these parameters can be fixed based on biologically reasonable assumptions and kinetic data available in the literature.

The half life of several unmodified DUSP proteins were reported as being between 20 and 45 min (DUSP1, 4, 5, 6, 16). However, following posttranslational regulation by phosphorylation and ubiquitination, these half lives ranged from as short as 7.5 min to as long as 4 h (Katagiri et al., 2005; Kucharska et al., 2009; Cagnol and Rivard, 2012). The half live of DUSP6 mRNA was reported as being between 20 and 40 min, which decreased to as little as 8 min following the inhibition of basal MEK activity (Bermudez et al., 2011). Neglecting this complexity, the model assumes equal half lives for protein and mRNA at a value of 30 min for all phosphatases, which fixes the degradation parameters according to *k*_deg_ = *p*_deg_ = *log*(2)/(30 min). Further, the model assumes a 10-fold amplification from the mRNA level to the protein level, fixing the protein synthesis parameter according to *p*_synt_ = 10*p*_deg_. The expression level of mRNA was normalized (*x* ≤ 1), which fixes the mRNA synthesis parameter at *p*_synt_ = *k*_deg_. The remaining parameters were chosen such that for strong, constant activity of the upstream kinase yields reasonable expression levels of around 80% (*x* ≈ 0.8) of the maximal possible value. Further assuming a reasonable degree of ultrasensitivity *n* = 2, fixes the threshold of half activation at *K* = 0.5.

## Conflict of Interest Statement

The authors declare that the research was conducted in the absence of any commercial or financial relationships that could be construed as a potential conflict of interest.

## Research Topic:

From structural to molecular systems biology: experimental and computational approaches to unravel mechanisms of kinase activity regulation in cancer and neurodegeneration

## Topic Editor:

Matteo Barberis, Humboldt University Berlin, Germany; Max Planck Institute for Molecular Genetics, Berlin, Germany
